# Correction: *Readiness of public health facilities to provide quality maternal and newborn care across the state of Bihar, India: a cross-sectional study of district hospitals and primary health centres*

**DOI:** 10.1136/bmjopen-2018-028370corr1

**Published:** 2019-08-30

Kaur J, Franzen SRP, Newton-Lewis T, *et al*. Readiness of public health facilities to provide quality maternal and newborn care across the state of Bihar, India: a cross-sectional study of district hospitals and primary health centres. *BMJ Open* 2019;9:e028370. doi: 10.1136/bmjopen-2018-028370

This article was previously published with an error.

The initials of Georgina Murphy were missing. The correct author name is Georgina A V Murphy.

The correct affiliation of Georgina A V Murphy is Centre for Tropical Medicine and Global Health, University of Oxford, Oxford, UK.

Samuel Richard Piers Franzen is affiliated only to Oxford Policy Management, Oxford, UK.

The other two affiliations are not applicable to any of the authors:

^4^Health Services Unit, Kenya Medical Research Institute/ Wellcome Trust Research Programme, Nairobi, Kenya

^5^Bill and Melinda Gates Foundation, Seattle, Washington, USA

