# Supplementary material for: Readiness of public health facilities to provide quality maternal and newborn care across the state of Bihar, India: a cross-sectional study of district hospitals and primary health centres
Source: BMJ Open. 2019 Jul 29;9(7):e028370. doi: 10.1136/bmjopen-2018-028370 (PMC6678016; doi:10.1136/bmjopen-2018-028370)
Supplement: Supplementary data [file bmjopen-2018-028370supp001.pdf]

APPENDIX 1

Additional methods

Infrastructure scoring

A score out of 1 was generated for each of these components. These scores were added, giving equal weight to each component and divided by the total components to generate an overall score ranging from 0 to 1, which was then converted to a percentage by multiplying by 100.

To compute a score for the component 'power/electricity' in the facilities, a value 1 was assigned to the facilities with continuous power, 0.75 to the facilities which reported occasional disruption to power in summer only, 0.5 to the facilities that were facing occasional disruption to power throughout the year, 0.25 to facilities with regular interruption to power and 0 to the facilities that reported no power supply. In cases where back-up was available that met the entire needs of the facility the above score was replaced by 1. For the 'water' score, 0.5 was assigned for availability of 24*7 running water and 0.5 if filtered water was available to patients. For telephone connection, a value 1 was assigned if it was available, else 0.

The transport score was computed by giving a score 0.5 to availability of at least one functional ambulance and an additional 0.5 to 24*7 availability. For toilets, 0.5 was assigned in case a functional toilet was available and 0.25 in case a toilet was available but not functional. Another 0.5 was assigned if the condition of the toilet was good and 0.25 if it needed repair/maintenance. For handwashing stations, 1 was assigned for the availability for elbow tap with running water and 0.5 was assigned for tap (without elbow) with running water. For computer and internet, 0.5 was assigned if the hospital had provided a computer and an additional 0.5 if there was internet connection available at the facility. For blood bank, 1 was assigned if there was a blood bank provision at the hospital, else 0.

For room availability, a score of 1 was assigned for the availability of each of the 16 rooms and then, it was added across rooms and divided by 16 to get a score out of 1. The 16 rooms were waiting room, labour room, maternity ward, immunization room, counselling room, operation theatre, store room, paediatrics, labour room eclampsia, SNCU (sick new born care unit), NBSU (new born stabilization units), septic labour room, antenatal ward, postnatal ward, postpartum ward and post-operative ward.

Equipment scoring

To compute the equipment score, a value 1 was assigned to each equipment if it was available and functional, else it was assigned 0. After computing the score for each equipment, we divided it by 48 (total equipment) to get a score out of 1 and multiplied by 100 to get the percentage distribution.

Supplies/drugs scoring

The availability of a drug was assigned a value 1, else it was assigned 0. The scores for each of the drugs were added, divided by the total (76) and then multiplied by 100 to get the percentage distribution.

Table S1: Infrastructure assessed

Infrastructure Components	Facility Level
Handwashing in immunization Room	PHCs, DHs
Handwashing in laboratory	PHCs, DHs
Handwashing in operation theatre	PHCs, DHs
Toilet in labour room	PHCs, DHs
Handwashing in labour room	PHCs, DHs
Transport	PHCs, DHs
Water	PHCs, DHs
Power	PHCs, DHs
Phone	PHCs, DHs
Room availability	DHs
Blood bank	DHs
Computer and internet	DHs

Abbreviations: PHC: Primary health centre, DH: District hospital

Table S2: Equipment assessed in district hospitals and primary health centres

Equipment
Caserean kit
Neonatal stethoscope
MTP suction
Light examination
NSV kit
Infantometer
Baby incubator
MTP kit
Nebulizer
Feeding tube
Shadowless lamp (OT)
Conventional vasectomy kit
Oxygen cylinder (LR)
Minilap kit
Resuscitation kit
Spotlight (OT)
Autoclave (NBCC)
Stethoscope (OT)
IV stand (OT)
Step up stool
Immunization table
Oxygen cylinder (OT)
Hub cutter (immunization room)
Partograph
Phototherapy unit

Cord clamp
Thermometer (LR)
IV cannula
Hub cutter (NBCC)
PPIUCD kit
Pump suction
Autoclave (OT)
Freeze tags
Stethoscope (LR)
Sterile gloves
Radiant warmer
Thermometer (immunization room)
Delivery kit
IUCD kit
Refrigerator
Ambu bag (NBCC)
Deep freezer
Operating table
Vaccine Carrier
BP Instrument
Labour table
Baby weighing machine
Ice packs

Information in parantheses represent the room in which the equipment was checked.
Abbreviations**:** MTP: Medical termination of pregnancy; NSV: Non-scalpel vasectomy; OT: Operation theatre; LR: labour room; NBCC: Newborn care corner; PPIUCD: Postpartum Intrauterine Contraceptive Device; IUCD: Intra-Uterine Contraceptive Devices; BP: Blood pressure

Table S3: Drugs assessed in district hospitals and primary health centres

Drugs
Amitriptyline
Captopril
Suxamethonium bromide
Mephenteramine
Carboprost
Miltefosine
Syrup salbutamol
Hydralazine
Omeprazole
Chlorine solution
Halothane (inhalation)
Menadione (Vit K3)
IUCD 375
Methyldopa
Simvastatin
Amphotericin B
Liposomal amphotericin B
Phytonadione
Glibenclamide
Skin disinfectant
Vitamin K
Thiopental (powder)
Dopamine
OPV
Deriphylline
BCG
Hepatitis B
DPT
Pentavalent
Frusemide
Measles
Epinephrine
Syrup vitamin A
Iron folic acid – small
Atenolol
Pentazocin chloride
Ceftriaxone
Methylegometrine (Methargine)
Nifedepine
Xylocard
Promethazine
Betamethasone
Hydrocortisone succinate

Antiseptic solution
Tetanus Toxoid
Pheneramine maleate
Adrenaline
Co-trimoxazole
Injection ranitidine
Zinc tablets
Syrup paracetamol
IUCD 380A
DEC (Diethyl Carbamazine)
Iron folic acid – Large
Amoxicillin
Metoclopramide
OCPs (Mala-N)
Ampicillin
Calcium gluconate
ECPs
Lignocaine hydrochloride 2%
Misoprostol
Condoms (Nirodh)
Ciprofloxacin
Diclofenac
Magnesium sulphate
Gentamycin
Atropine
Oxytocin
Albendazole
Ketamine
Diazepam
ORS
Dexamethasone
Metronidazole
Paracetamol

Abbreviation: IUCD: Intra-Uterine Contraceptive Devices; BCG: Bacille Calmette Guerin; DPT: Diphtheria, Pertussis and tetanus; OPV: Oral Polio Vaccine; ORS: Oral Rehydration Solution; OCP: Oral contraceptive pill; ECP: Emergency contraceptive pill

Table S4: Essential and Desirable Staff Guidelines at district hospitals (DHs) and primary health centres (PHCs) as per IPHS Guidelines

A: PHCs

IPHS Guidelines	Type A		Type B	
Staff	Essential	Desirable	Essential	Desirable
Medical Officer-MBBS	1		1	1
Medical Officer-AYUSH		1		1
Pharmacist	1		1	
Nurse-midwife (ANMs)	3	1	4	1
Laboratory Technician	1		1	

Type A PHC: PHC with delivery load of less than 20 deliveries in a month and Type B PHC: PHC with delivery load of 20 or more deliveries in a month

B: DHs

IPHS Requirements	100 Beds	200 Beds	300 Beds	400 Beds	500 Beds
Medical officer	11	13	15	19	23
Staff Nurse/ANMs	45	90	135	180	225
Lab Technician	6	9	12	15	18
Compounder/Pharmacist	5	7	9	11	13
Store Keeper	1	1	2	2	2

Table S5: Number of respondents at district hospitals (DHs) and primary health centres (PHCs).

No. of respondents at DHs	No. of respondents at PHCs	Information covered
36	190	Overall Infrastructure, Staffing
35	171	Laboratory: Infrastructure
36	179	Labour room: Infrastructure, Equipment, Supplies
35	181	Immunization room: Infrastructure, Equipment
36	166	Operation Theatre: Infrastructure, Equipment
35	177	Drug Store room: Supplies

Note: This table shows the number of respondents that were available for each of these components. The information on sub-components could vary based on the knowledge of the respondent.

Table S6: Reasons for unavailability of services at district hospitals (DHs) and primary health centres (PHCs)

	% Unavailable		*Reasons for unavailability*							
			Infrastructure		Equipment		Drugs		Supplies	
Services	DHs	PHCs	DHs	PHCs	DHs	PHCs	DHs	PHCs	DHs	PHCs
MTP	28%	81%	3%	23%	6%	37%	0%	17%	28%	71%
IUCD insertion	0%	3%	0%	1%	0%	1%	0%	0%	0%	2%
IUCD removal	0%	3%	0%	1%	0%	1%	0%	0%	0%	2%
Post-partum IUCD insertion	0%	16%	0%	6%	0%	7%	0%	3%	0%	13%
Non-scalpel vasectomy	11%	70%	3%	16%	6%	31%	3%	10%	6%	61%

Conventional vasectomy	19%	42%	3%	10%	3%	15%	0%	2%	6%	35%
Minilap tubectomy	3%	8%	3%	2%	0%	2%	0%	0%	3%	7%
Post-partum minilap sterilization	3%	28%	0%	7%	3%	12%	0%	4%	3%	22%
Laparoscopic sterilization	92%	95%	19%	23%	47%	50%	3%	13%	61%	81%
Distribution of condoms	6%	3%	0%	0%	0%	0%	3%	1%	0%	2%
Distribution of OCPs	3%	3%	0%	0%	0%	0%	3%	1%	0%	2%
Distribution of ECPs	11%	6%	0%	1%	0%	0%	11%	2%	0%	3%
Normal delivery	0%	5%	0%	2%	0%	2%	0%	1%	0%	3%
Caesarian section	3%	-	3%	-	3%	-	0%	-	0%	-
Managing complicated pregnancies	0%	31%	0%	10%	0%	17%	0%	5%	0%	24%
Managing delivery cases with eclampsia	8%	20%	0%	4%	0%	10%	0%	5%	8%	14%
Registration of pregnancies	0%	2%	0%	0%	0%	1%	0%	0%	0%	1%
Antenatal care visits	0%	2%	0%	0%	0%	1%	0%	0%	0%	1%
Distribution of IFA tablets	0%	5%	0%	0%	0%	1%	0%	4%	0%	1%
Tetanus toxoid vaccinations	3%	1%	3%	0%	0%	1%	0%	0%	0%	1%
VDRL tests during antenatal visits	17%	30%	3%	6%	11%	17%	3%	8%	3%	16%
BP tests during antenatal visits	0%	1%	0%	0%	0%	1%	0%	0%	0%	1%
HB tests during antenatal visits	0%	8%	0%	1%	0%	5%	0%	4%	0%	5%
Weighing of newborns at birth	0%	4%	0%	2%	0%	2%	0%	1%	0%	3%
Neonatal resuscitation in cases with asphyxia	3%	7%	3%	3%	0%	5%	0%	2%	3%	6%
Post-partum check ups within 48hrs of delivery	0%	5%	0%	2%	0%	2%	0%	1%	0%	4%
Post-partum check ups b/w 48hrs &14 days of delivery	0%	11%	0%	3%	0%	3%	0%	1%	0%	6%
Immunization sessions	53%	2%	3%	0%	6%	1%	6%	0%	6%	2%
Treatment for diarrhea	0%	3%	0%	1%	0%	2%	0%	1%	0%	2%
Treatment for acute respiratory infections	8%	23%	3%	7%	3%	8%	0%	5%	3%	20%

Abbreviations: MTP: Medical termination of pregnancy; IUCD: Intra-Uterine Contraceptive Devices; OCP: Oral contraceptive pill; ECP: Emergency contraceptive pill; IFA: Iron folic acid

Table S7: Most commonly missing and available structural capacity items across primary health centres and district hospitals

	Infrastructure		Equipment		Drugs	
	**Most commonly missing**	**Most commonly available**	**Most commonly missing**	**Most commonly available**	**Most commonly missing**	**Most commonly available**
**Primary health centres**	Handwashing in immunization room (13%)	Water (81%)	Neonatal stethoscope (16%)	Deep freezer, Ambu bag (97%)	Amitriptyline (12%)	ORS (95%)
	Handwashing in laboratory (37%)	Power (87%)	MTP suction (17%)	Operating table, labour table, BP Instrument, vaccine carrier (98%)	Captopril (14%)	Dexamethasone (99%)
	Handwashing in OT (60%)	Phone (93%)	Light examination (20%)	Baby weighing machine, ice packs (99%)	Suxamethonium bromide, mephenteramine (16%)	Paracetamol, metronidazole (100%)
**District hospitals**	Handwashing in immunization room (19%)	Water (97%)	Infantometer, nebulizer, baby incubator (14%)	IV cannula, PPIUCD kit, ice packs (94%)	Captopril, phytanodiane, simvastin, IUCD 35 (3%)	Atropine, DPT (94%)
	Handwashing in laboratory (47%)	Power (99%)	Neonatal stethoscope, MTP suction (39%)	Vaccine carrier, operating table, delivery kit, oxygen cylinder (97%)	Amritriptyline (6%)	Dexamethasone, metronidazole, paracetamol, BCG, OPV, measles, pentavalent (97%)
	Room Availability (64%)	Transport (100%)	Light examination, resuscitation kit, phototherapy unit (42%)	Labour table, MTP kit, IUCD kit, Ambu bag, IV stand (100%)	Glibenclamide, suxamethonium bromide, syrup vit A (8%)	Diazepam, tetanus toxoid (100%)
