# Supplementary material for: Readiness of public health facilities to provide quality maternal and newborn care across the state of Bihar, India: a cross-sectional study of district hospitals and primary health centres
Source: BMJ Open. 2019 Jul 29;9(7):e028370. doi: 10.1136/bmjopen-2018-028370 (PMC6678016; doi:10.1136/bmjopen-2018-028370)
Supplement: Author's manuscript [file bmjopen-2018-028370.draft_revisions.pdf]

BMJ Open is committed to open peer review. As part of this commitment we make the peer review history of every article we publish publicly available.

When an article is published we post the peer reviewers' comments and the authors' responses online. We also post the versions of the paper that were used during peer review. These are the versions that the peer review comments apply to.

The versions of the paper that follow are the versions that were submitted during the peer review process. They are not the versions of record or the final published versions. They should not be cited or distributed as the published version of this manuscript.

BMJ Open is an open access journal and the full, final, typeset and author-corrected version of record of the manuscript is available on our site with no access controls, subscription charges or pay-per-view fees (http://bmjopen.bmj.com).

If you have any questions on BMJ Open's open peer review process please email info.bmjopen@bmj.com

BMJ Open

Readiness of public health facilities to provide quality maternal and newborn care across the state of Bihar, India: a cross-sectional study of district hospitals and primary health centres

Journal:	*BMJ Open*
Manuscript ID	bmjopen-2018-028370
Article Type:	Research
Date Submitted by the Author:	10-Dec-2018

Complete List of Authors:	kaur, Japneet; Oxford Policy Management,<br>Franzen, Samuel; Oxford Policy Management, ; University of Oxford, Centre for Tropical Medicine and Global Health<br>Newton-Lewis, Tom; Oxford Policy Management<br>Murphy, Georgina ; Kenya Medical Research Institute/Wellcome Trust Research Programme, Health Services Unit; Bill and Melinda Gates Foundation

Keywords:	Health & safety < HEALTH SERVICES ADMINISTRATION & MANAGEMENT, Health economics < HEALTH SERVICES ADMINISTRATION & MANAGEMENT, Health policy < HEALTH SERVICES ADMINISTRATION & MANAGEMENT, Quality in health care < HEALTH SERVICES ADMINISTRATION & MANAGEMENT

SCHOLARONE™
Manuscripts

Full Title: Readiness of public health facilities to provide quality maternal and newborn care across the state of Bihar, India: a cross-sectional study of district hospitals and primary health centres

Short title: Readiness of health facilities to provide quality maternal and newborn care in Bihar, India

Authors: Japneet Kaur* [1], Sam Frazen[2], Tom Newton-Lewis[1], Georgina A. V. Murphy[1,3]

Affiliations:

1.Oxford Policy Management(OPM), Delhi, India

2. Oxford Policy Management(OPM), Oxford, UK

3. Centre for Tropical Medicine and Global Health, University of Oxford, UK

***Corresponding author:** Japneet Kaur, Oxford Policy Management, 4/6 First Floor, Siri Fort Institutional Area, New Delhi, India Email: japneetkaur58@gmail.com. Tel: (+91) 011-48081111

Key words: neonatal care, maternal care, newborn, infrastructure, newborn mortality, maternal mortality, district hospital, primary health care, audit, staffing, quality of care, Bihar, India

Abstract *(max 300 words)*

Introduction: Poor access to quality healthcare is one of the most important reasons of high maternal and neonatal mortality in India, particularly in poorer states like Bihar. India has implemented initiatives to promote institutional maternal deliveries. It is important to ensure that health facilities are adequately equipped and staffed to provide quality care for mothers and newborns.

Methods: We conducted a cross-sectional study of 190 primary health centres (PHCs) and 36 district hospitals (DHs) across all districts in Bihar to assess the readiness of facilities to provide quality maternal and neonatal care. Infrastructure, equipment and supplies, and staffing were assessed using the WHO service availability and readiness assessment and Indian public health standard (IPHS) guidelines. Additionally, we used household survey data to assess the quality of care reported by mothers delivering at study facilities.

Results: PHCs and DHs were found to have 61% and 67% of the mandated structural components to provide maternal and neonatal care, on average, respectively. DHs were, on average, slightly better equipped in terms of infrastructure, equipment and supplies by comparison to PHCs. DHs were found to be inadequately prepared to provide neonatal care. Lack of recommended handwashing stations and bins at both DHs and PHCs suggested low levels of hygiene. Only half of the essential drugs were available in both DHs and PHCs. While no association was revealed between structural capacity and patient-reported quality of care, adequacy of staffing was positively associated with the quality of care in DHs.

Conclusion: Examining all DHs and a representative sample of all PHCs in Bihar, this study revealed that improvement in service readiness is essential, to provide quality care to mothers

For peer review only - http://bmjopen.bmj.com/site/about/guidelines.xhtml

and newborns. Access to quality care is essential if progress in reducing mortality is to be achieved in this populous high-burden state.

Strengths and limitations of this study

- The findings of this study are based on data collected from a single visit to these facilities; the availability of different equipment and supplies might vary over time.
- There is incomplete data in some facilities. Therefore, the number of responses varied across and within the components of infrastructure, supplies, equipment and staffing.
- With respect to household data, women providing information on quality of care were not representative of those delivering at facilities and the sampling was not proportional to the number of deliveries at each facility

INTRODUCTION

Progress has been made in reducing maternal and newborn mortality in India over the last three decades. From 556 per 100,000 and 57 per 1,000 live births in 1990, the maternal and neonatal mortality in India reduced to 174 per 100,000 and 26 per 1,000 in 2015, respectively.[1] However, considerable further improvements will be needed if India is to reach the Sustainable Development Goal of reducing maternal mortality to less than 70 per 100,000 births and neonatal mortality to at least as low as 12 per 1,000 live births by 2030.[2]

These goals will be particularly challenging for Bihar, the third most populated state in India (approximately 104 million). Bihar struggles with persistent poverty (34% of the population lives below the poverty line) and poor health outcomes (neonatal mortality rate of 27 per 1,000 live births and maternal mortality rate of 208 per 100,000 live births).[3–5] Only 63% of the pregnant women deliver in a health facility in Bihar, which is 12% lower than the national average.[6,7] This is an important area that needs attention since the biggest gains in survival are estimated to be achieved through facility-based maternal care provided at the time of childbirth and the immediate postpartum period along with newborn care.[8]

In recognition of the importance of facility-based maternal and newborn care, India has implemented many initiatives to encourage institutional deliveries. The most ambitious of these is the Janani Suraksha Yojana (JSY) programme offering conditional cash transfers to women of low socio-economic status for delivering at a health facility.[9] Despite the success of JSY in increasing institutional deliveries, gaps in the readiness of facilities to provide quality care have been highlighted as an important barrier for the programme to have the intended effect on health outcomes.[10,11]It is crucial to ensure that facilities are adequately resourced and equipped to deliver essential maternal and new born care.[12–15] At the minimum, health facilities require adequate infrastructure, equipment, supplies, and skilled staff if significant improvements in reducing mortality and morbidity are to be achieved.

The public health system in India comprises of a three-tier system, namely, primary care at the village level, secondary care at the sub-district and district levels and tertiary levels of health care at the regional level. The district hospital (DH) is an essential medium of secondary level of health care with an objective to provide curative, preventive and promotive health care services to the people in the district. Linked to every DH are health centres providing primary care, including sub-

For peer review only - http://bmjopen.bmj.com/site/about/guidelines.xhtml

divisional hospitals, community health centres (CHCs), primary health centres (PHCs) and sub-centres. PHCs are crucial to the health system as they form the first point of contact to a qualified doctor of the public sector for the patients. There are two kinds of PHCs, one is called additional PHC which mainly does clinical work and the other is block PHC which also exercises administrative powers in the entire block. Serving a population between 20,000 and 30,000, PHCs act as a referral unit for six sub-centres and refer out cases to CHCs and higher order facilities. In Bihar, there are 36 DHs, 70 CHCs, 9729 sub-centres and 1883 PHCs (including 534 block PHCs).[16]

This study aims to assess structural and staffing gaps in the public health facilities, specifically, PHCs and DHs in Bihar, to deliver essential maternal and newborn services. This study is based on the data collected in the baseline assessment of Bihar Technical Support Programme (BTSP). BTSP is a large multi-year programme funded by the Bill and Melinda Gates Foundation and implemented by CARE India with OPM as monitoring and evaluation partner.[17] Working closely with Government of Bihar's Departments of Health and Family Welfare and Social Welfare, CARE India's interventions aim to strengthen the health system and improve the quality of care to improve reproductive, maternal, newborn, child, adolescent and nutrition (RMNCH+N) outcomes.

METHODS

We conducted a cross-sectional study of health facilities in Bihar during July-October 2016. Facility surveys were conducted in block (sub-district) and district level government-run public health facilities. This study also uses household maternal and child health survey data collected during October-December 2016 by CARE India.

Study population and sampling

There are 36 district hospitals in Bihar, all of which were invited to participate in the facility survey. There are 534 blocks (sub-districts) in Bihar, 190 of which were sampled for the facility survey. Blocks were sampled proportionally according to the total number of blocks per district, resulting in 5-6 blocks sampled per district. Each block contains one block PHC, all of which (from the 190 sampled blocks) were included in the facility survey.

Household survey data were collected from mothers who had a child belonging to the following five age groups: i) 0-2; ii) 3-5; iii) 6-8; iv) 9-11; and v) 12-23 months old. A mixed sampling methodology of population based-estimation and lot quality assurance sampling (LQAS) (a small sample survey design based on binomial distribution) was used [18]. The sampling 'lots' in this survey were the blocks/sub-districts. All 534 blocks in 38 districts were sampled. The number of Anganwadi Centers (AWC, village level institutions providing basic health care services) sampled from each block was determined using proportional allocation. The sampled AWC were selected within each block using simple random sampling. Within each sampled AWC catchment area, households were identified through systematic sampling.[18] Briefly, an index household was chosen within each AWC catchment area using a random number table. Starting with the index household, data collectors visited every 10th household looking for eligible mothers. The data collectors continued moving in a circular manner, following the 'right-hand rule', until five interviews were conducted with eligible mothers (one from each age group).Only data collected from mothers with children aged between 0-2 months and who also delivered at the DHs or PHCs

For peer review only - http://bmjopen.bmj.com/site/about/guidelines.xhtml

that were covered in the facility survey (ranging from 1 to 17 mother per facility) were included in this analysis.

Data collection

Facility survey

Data were collected using a standardised structured survey tool designed based on the Service Availability and Readiness Assessment (SARA) tool developed by the World Health Organisation (WHO) and the United States Agency for International Development.[19] The tool was modified for the Indian context using the Indian Public Health Standards (IPHS) guidelines.[20,21] To evaluate the structural capacity of the facility, the availability and condition of infrastructure, equipment, and supplies in different departments, including the labour room, New Born Care Corner (NBCC), immunization room, laboratory, operation theatre, drug store, and data operation were assessed. Information on infrastructure and equipment was collected through interviews with the facility-in-charge and staff nurse as well as through direct observation. The pharmacist or drug store-in-charge was interviewed and the responses were validated through the drug register to collect information on supplies availability.

The Medical Officer in Charge (MOIC) at the PHCs and Hospital Manager at the DHs were also interviewed to obtain information on the number of health personnel employed at the facilities and the number of personnel that were sanctioned (number of staff expected to be employed) to the facilities for each of the health cadres, including medical officers (MOs), staff nurses, auxiliary nurse midwifery(ANMs), laboratory technicians, and pharmacists. This information was also cross-checked with the facility registers.

Availability of 30 services related to family planning, safe delivery, antenatal care, and neonatal and child care was assessed and the reasons for unavailability were asked from the MOIC in PHCs and the Hospital Manager in DHs.

Three pilot tests were conducted in the facilities outside the study sample to refine the survey tool and to train the enumeration team. The survey was conducted by 60 enumerators over the four month period. Enumerators all had prior experience in conducting facility surveys and received further training over 10 days on using the study tool and conducting this survey.

Periodic data checks for completeness and outliers were conducted by a data management team in Patna, Bihar. Where information was missing due to absenteeism or lack of time provided by the respondent, a second visit to those facilities was organized.

LQAS Household survey

One-to-one interviews were conducted with consenting and eligible mothers by trained data collectors, using a standardised questionnaire and following standard operating procedures. Information collected from mothers and of interest to this study included the household characteristics, the place of delivery, and care received at the place of delivery.

Patient and Public involvement

For peer review only - http://bmjopen.bmj.com/site/about/guidelines.xhtml

Mothers with children belonging to the following five age groups: i) 0-2; ii) 3-5; iii) 6-8; iv) 9-11; and v) 12-23 months old were interviewed. Information with respect to the household characteristics, the place of delivery, and care received at the place of delivery was collected from the mothers to complement our findings of the readiness of the facilities to provide quality care.

Data Analysis

Data analysis was conducted using Stata version 13 (Stata Corporation, USA). The current status of the facilities was assessed on three broad parameters, namely, the structural capacity, staffing, and the quality of care provided at the facilities.

Structural Capacity

The structural capacity of the facilities was assessed by computing readiness scores of 0-1 for infrastructure, equipment and supplies using a set of questions included in our facility assessment tool. "Infrastructure readiness" included the availability as well as the condition of different components, wherever applicable. For equipment, "readiness" implied the availability as well as functionality of the equipment and for supplies, readiness was defined by availability.

Infrastructure readiness of the facilities included nine broad components (such as power, water, transport, handwashing stations) at the PHCs.[19] An additional three components (availability of different rooms, computer and internet, and blood bank) were assessed for DH infrastructure score (details of components are listed in **appendix table S1**).

The equipment readiness of the facilities was assessed by scoring the availability and functionality of 48 essential (according to IPHS guidelines) maternal and newborn health equipment (items listed in **appendix table S2**). A score of 1 was assigned if the equipment was observed to be available and in a functional state. In case of unavailability or available but not functional equipment, a score of 0 was assigned. Similarly, supplies readiness was assessed by considering the availability of 76 essential maternal and child health drugs that were expected at the facilities as per the IPHS guidelines (listed in **appendix table S3**). Detailed methods of scoring have been provided in **the appendix**.

Staffing Index

We assessed the availability of human resources by computing the ratio of filled to sanctioned positions, as reported by the MOIC and the Hospital Manager or equivalent authority in charge in the PHCs and DHs, for each health cadre in each facility. The ratio of total filled to total sanctioned positions for permanent staff, combining all cadres, was computed to generate an overall staffing index for each facility.

The availability of health staff was also compared with the essential requirements mandated by IPHS guidelines. In PHCs, we considered staff requirement based on the monthly delivery load of more than 20, as provided by the IPHS guidelines.[20] In DHs, the staff requirement based on the bed strength were rounded down to compare with the mandated guidelines.[21] For instance, for DHs with less than or equal to 200 beds, we considered the staff requirements for 100 beds

For peer review only - http://bmjopen.bmj.com/site/about/guidelines.xhtml

as defined by IPHS guidelines. For ANMs, the IPHS requirement of 0.45 staff per bed was considered. **(appendix table S4)**.

Quality of care

Our primary aim was to describe the structural readiness of facilities to provide essential maternal and new-born services. We also conducted analyses of household survey data to explore the quality of care at facilities as reported by women who both participated in the household survey and delivered at study facilities.

Each mother was asked 11 questions during the household survey pertaining to the treatment and care that they and their newborns received during delivery. Each question was assigned a score of 0 (not performed/don't know) or 1 (performed). A quality of care index for each PHC and DH was generated by taking the average score of the 11 questions for all those household survey participants who delivered within the facility.

The relationship between structural capacity, staffing, and quality of care indexes were visually explored using scatter plots and trend lines as part of this exploratory analysis.

Ethics and permission

Ethical approval was granted by the Indian Institutional Review Board. Oral consent to conduct this study was obtained from the MOIC and the Hospital Manager or equivalent authority in charge in the PHCs and DHs, respectively. For the household survey, ethics approval was obtained from Ashirwad Ethics Committee, Ashirwad Hospital and Research Center, Ulhasnagar, India.

RESULTS

Facility survey data were collected from a total of 190 PHCs and 36 DHs. The number of facilities assessed for each component of structural capacity and staff availability varied (range: 35-36 DHs and 166-190 PHCs) due to missing data and depending on the availability of respondents during the time of the survey **(appendix table S5).** Household survey data were available from 671 mothers who delivered in 107 of the 190 study PHCs and 1419 mothers who delivered in one of the 36 study DHs.

Facility characteristics

Most PHCs (95%) were functional for 24 hours per day, but 40% of them were not accessible throughout the year. A dedicated labour room, maternity ward, operation theatre and store room was available in most PHCs (94%, 96%, 89% and 96% respectively); an immunisation room was available in only 76% of the PHCs. While the IPHS guidelines recommend each PHC to have six beds, the number of sanctioned and available beds, as reported by the MOIC, varied. There were 12 beds on average per PHC but eight PHCs reported having no beds, four of which nonetheless conducted maternal deliveries.

For peer review only - http://bmjopen.bmj.com/site/about/guidelines.xhtml

All DHs had a dedicated labour room and maternity ward, but specialised units for antenatal care and for post-natal care were available in only 69% and 56% of the DHs, respectively. A dedicated room for counselling was provided in 67% of the DHs. As per the IPHS guidelines, every DH should have a provision for Special New Born Care units (SNCUs); however, this unit was found in only 21 of the 36 DHs (58%). In DHs, the number of beds recommended by IPHS guidelines varies between 75 to 500 depending on the size, terrain, and population of the district; however, in Bihar, we identified four DHs with fewer than 75 beds available.

Availability of services

Of the 30 services assessed in 36 DHs and 189 PHCs, seven (23%) and 12 (40%) services were unavailable in at least 10% of the facilities, respectively. Most of the commonly unavailable services were related to family planning including medical termination of pregnancy (MTP), non-scalpel vasectomy, conventional vasectomy, and laparoscopic sterilization. Venereal disease research laboratory (VDRL) tests conducted during ANC visits were unavailable in 17% and 30% of the DHs and PHCs, respectively (**figure 1).**

For both PHCs and DHs, the main reason for the lack of these services was reported to be lack of required human resources (**figure 1** and **appendix table S6**). In PHCs, lack of equipment was reported to be the second most important factor for the unavailability of services such as MTP, non-scalpel vasectomy and laparoscopic sterilization. Lack of equipment was also the reason for unavailability of laparoscopic sterilization in 47% of the 36 DHs.

For peer review only

For peer review only - http://bmjopen.bmj.com/site/about/guidelines.xhtml

Figure 1: Reasons for unavailability of services in (A) primary health centres (PHCs) and (B) district hospitals (DHs)

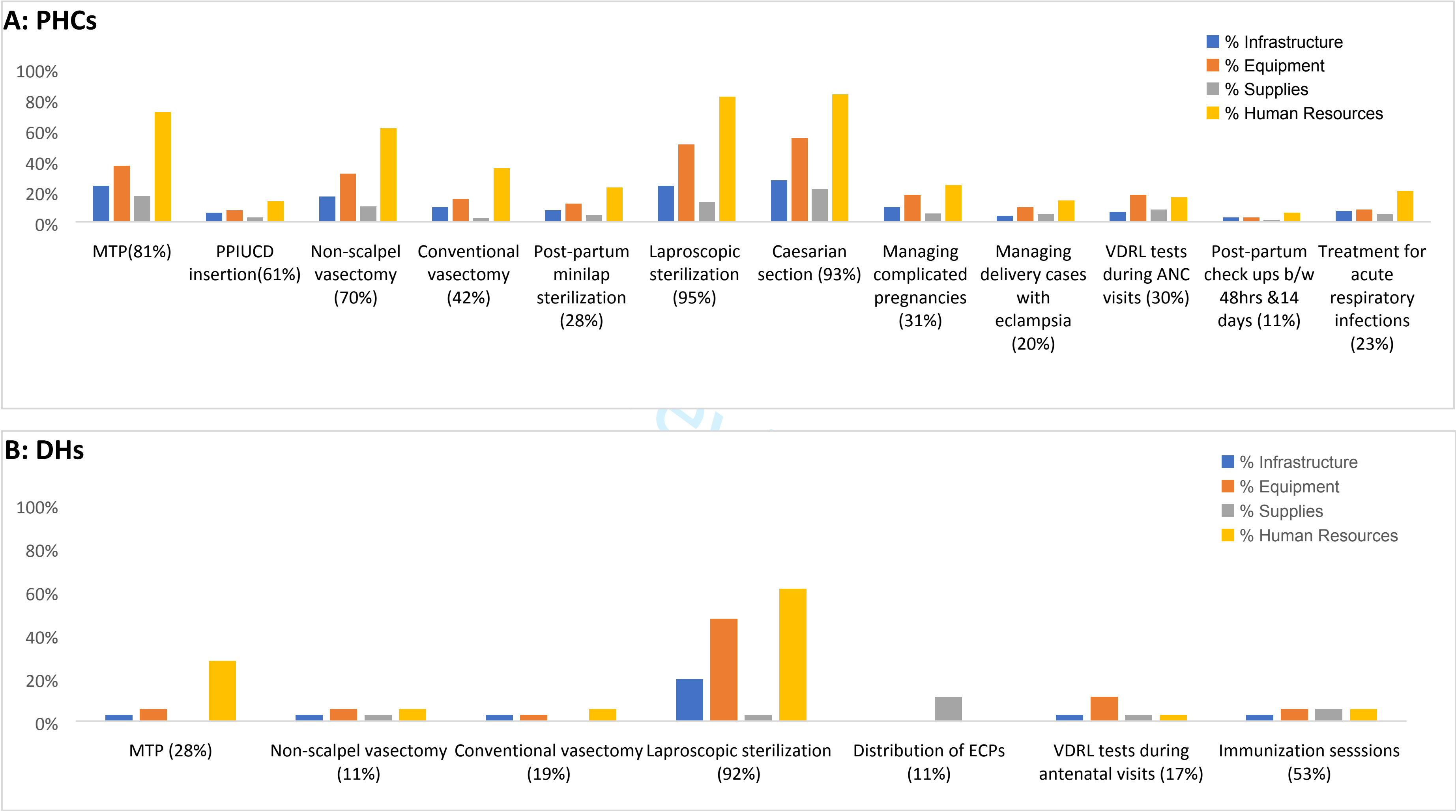

Multiple answers were allowed. Figures in parentheses of x axis report the total unavailability

Abbreviations: MTP: Medical termination of pregnancy; ECP: Emergency contraceptive pill; VDRL: Venereal Disease Research Laboratory; PPIUCD: Postpartum Intrauterine Contraceptive Device; ANC: Ante natal care

For peer review only - http://bmjopen.bmj.com/site/about/guidelines.xhtml

Structural capacity

Overall, the average structural capacity across PHCs was 61% (range: 36-85%) and 67% (range: 50-84%) across DHs. DHs were slightly better equipped on average in terms of availability of infrastructure, equipment and drug supplies (78%, 70%, 52%, respectively) compared with PHCs (67%, 65%, and 50%, respectively). Results varied greatly between facilities, particularly among PHCs (**figure 2**).

Figure 2: Structural readiness scores across district hospitals (DHs) and primary health centres (PHCs)

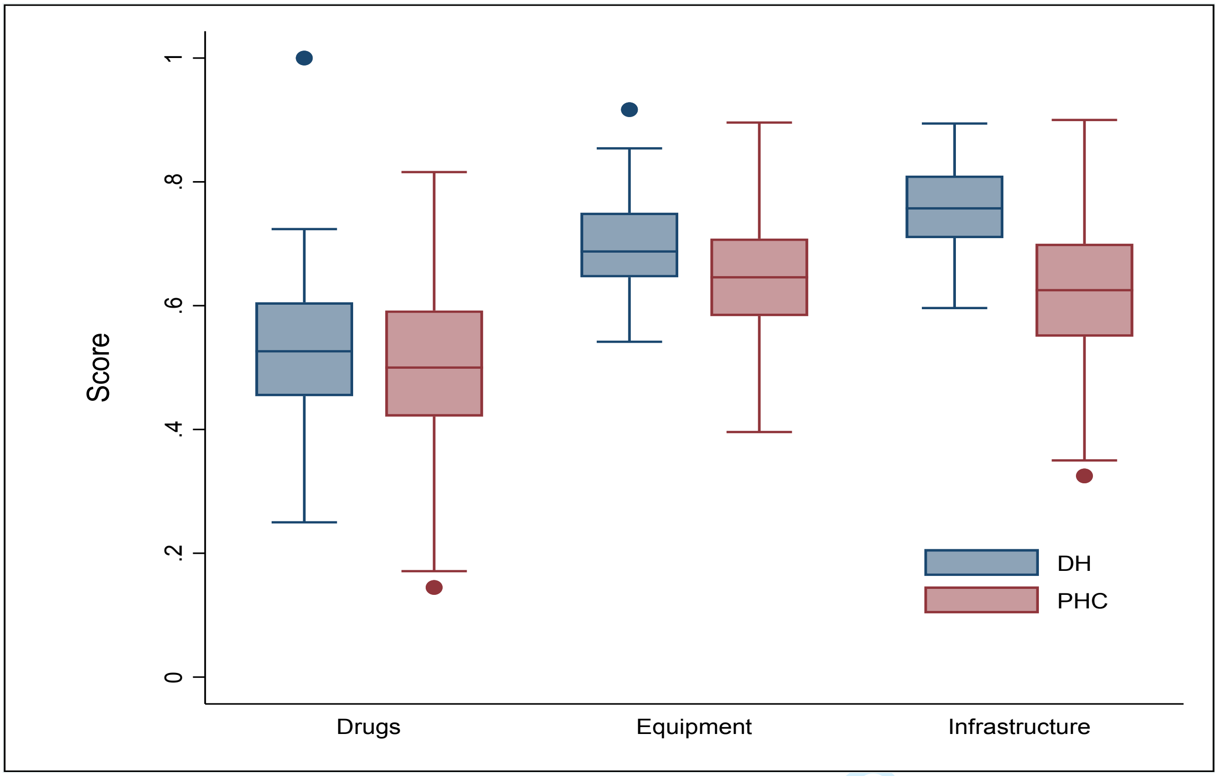

Scores are presented as box plots representing the median and interquartile range (box and whiskers, respectively) and outliers (dots).

Infrastructure

Of the 12 items assessed in DHs, five (hand washing station in the labour room, telephone connection, water, power and transport) had an average readiness score of greater than 90%. In PHCs, telephone connection was the only component with an average readiness score of over 90% across facilities. Readiness was particularly low with respect to hand washing stations in the immunisation room and laboratory in both DHs and PHCs (**appendix table S7**).

Of all the items accessed in the labour room, the availability of different colour coded bins to segregate waste into infectious and non-infectious sources was the lowest in both PHCs and DHs (54% and 63%, respectively). Emergency transport for referrals was available in only 66% of the PHCs, whereas the DHs performed well in this regard with all DHs having emergency transport available for referrals.

For peer review only - http://bmjopen.bmj.com/site/about/guidelines.xhtml

Equipment and supplies

Neonatal stethoscope and MTP suction were the two most commonly missing items of equipment in PHCs; whereas, in DHs, infantometer and nebulizer were the two most commonly missing items (**appendix table S7**). In the labour room specifically, light examination, feeding tube and oxygen cylinder were the most commonly missing items of equipment in both the DHs and PHCs.

Availability of drugs was the weakest performing area of structural assessment for both DHs and PHCs (figure 1), with only half (50% and 52% on average, respectively) of the essential drugs (n=76) being available. Of 25 essential drugs that should be available in the labour room as per the IPHS guidelines, 62% and 72% were available on average in PHCs and DHs, respectively. Carboprost, hydralazine and methyldopa were the least commonly available of the drugs in both the PHCs and DHs.

Staff availability

The overall average staffing index for the three main cadres including MOs, ANMs and pharmacists (permanent) was 69% (range: 11-100%) in PHCs, indicating 31% of health worker sanctioned positions, as reported by the MOIC, being unfilled. The average staffing index at PHCs was found to be the highest for the ANMs, indicating a high proportion of sanctioned positions being filled (**table 1**). However, the requirement of ANMs, as mandated by the IPHS guidelines, was fulfilled in only 42% of the PHCs **(table 1)**. The mandate of having at least one MO at a PHC was fulfilled at all PHCs. However, the sanctioned positions, as reported by the MOIC, varied and the average staffing index of available to sanctioned MOs was 70% for contractual (n =129) and 68% for permanent staff. The proportion of positions filled in PHCs was the lowest for laboratory technicians (27%). These technicians were, however, supplemented by contractual workers, for whom 92% of sanctioned positions were filled. Almost a third (30%) of the PHCs did not have any pharmacist. RMNCH+ counsellors were available in only four PHCs (2%) and family planning counsellors in six PHCs (3%). None of the PHCs had an infant and young child feeding counsellor.

In DHs, the overall staffing index for three cadres was 55% (range 24-100%). The staffing index amongst the health personnel in DHs was found to be similar to PHCs (**table 1**); the staffing index was also highest for ANMs (78%) and lowest (35%) for laboratory technicians in DHs. For ANMs, the IPHS requirement of 0.45 staff per bed was fulfilled in only 15% of the DHs **(table 1)**. The average staffing index for MOs was 52% and the requirement of essential MOs as per the IPHS guidelines was fulfilled in 53% of the DHs. Nearly 60% of the DHs had less than half of the sanctioned positions for MOs and nurses filled.

Table 1: Average filled/sanctioned positions for staff and IPHS requirement fulfillment for district hospitals (DHs) and primary health centres (PHCs)

Designation	Average filled to sanctioned- DHs	Avgerage filled to sanctioned- PHCs	% DHs fulfilling IPHS requirements	% PHCs fulfilling IPHS requirements
Medical officer	52% (34)	68% (190)	53% (34)	100% (190)

For peer review only - http://bmjopen.bmj.com/site/about/guidelines.xhtml

Staff Nurse	44% (33)	42% (48)		-
Auxiliary nurse midwife	78% (24)	81% (173)	15%(33)*	42%(173)
Laboratory technician	35% (32)	27% (148)	0%(32)	27%(148)
Compounder/pharmacist	56% (32)	63% (171)	16%(32)	70% (171)
Store keeper	58% (28)	57% (101)	61% (28)	57%(101)

*Only permanent positions are considered. Cases where information on sanctioned positions was missing were excluded. Medical officers include physicians, obstetricians, paediatricians and anaesthetists. PHC IPHS guidelines mention to appoint atleast 4 Nurse- Midwives. We consider at least 4 ANMs for each facility since the information for Staff Nurse is unavailable for most facilities. *DH IPHS guidelines mention the requirement for staff nurse/ANM combined and hence we consider the combined availability of staff nurse and ANM*

Reported quality of care

When asked if 11 essential pre- and post-partum services had been carried out, the responses were similar between DHs and PHCs (**table 2**). Almost all mothers reported that hygiene and newborn warmth practices of wearing gloves, wiping the baby dry and wrapping the baby were being practiced in both PHCs and DHs. Provision of skin-to-skin contact was reported by fewer than half of women, regardless of facility type. Measuring blood pressure and advising mothers about their and their baby's health before discharge were received by less than 30% of the mothers.

Table 2: Quality of care reported by mothers delivering at the primary health centres (PHCs) and district hospitals (DHs)

	PHCs (n =671)			DHs (n = 1419)		
**Quality of Care**	**Yes**	**No**	**Don't Know**	**Yes**	**No**	**Don't Know**
Was the baby wrapped in a clean cloth after birth?	97.91%	1.34%	0.75%	96.41%	1.20%	2.40%
Did this person wear gloves before conducting your delivery?	96.87%	1.64%	1.49%	95.49%	1.20%	3.31%
Was the baby wiped dry after delivery?	95.68%	2.53%	1.79%	93.31%	2.47%	4.23%
Was the baby weighed after delivery?	92.55%	3.73%	3.73%	88.94%	5.64%	5.43%
After delivery, was nothing applied to the cord?	91.36%	8.67%	0%	85.27%	14.73%	0%
Did the person wash hands with soap before conducting your delivery?	76.15%	3.73%	20.12%	73.50%	5.14%	21.35%
Was the baby placed on the mother's abdomen immediately after birth?	49.78%	42.92%	7.30%	40.03%	48.98%	10.99%
Were you advised by the nurse or anyone else to keep the baby naked on your chest, next to your skin?	35.77%	63.49%	0.75%	23.82%	75.26%	0.92%
Did you breastfeed your baby immediately after delivery?	24.29%	75.71%	0%	21.17%	78.48%	0.24%
Was any advice given to you regarding your health or your baby's health before you were discharged from the facility?	29.06%	70.94%	0%	18.60%	81.40%	0%
Was blood pressure measured after delivery, before discharge?	9.99%	90.91%	0%	8.67%	91.33%	0%

For peer review only - http://bmjopen.bmj.com/site/about/guidelines.xhtml

Relationship between quality indexes

No clear relationship between the facility structural capacity index (composite score for infrastructure, equipment, and drugs), the staffing index (ratio of sanctioned to filled positions), or the quality of care index (average score for 11 facility-based care services among women per facility) was found for PHCs. In DHs, no clear trend was observed between the structural capacity index and quality of care as well as staffing and structural capacity index. However, a positive relationship between the quality of care index and staffing index was evident (**figure 3**).

Figure 3: Structural capacity, staffing and quality of care relationship for primary health centres (blue) and district hospitals (orange)

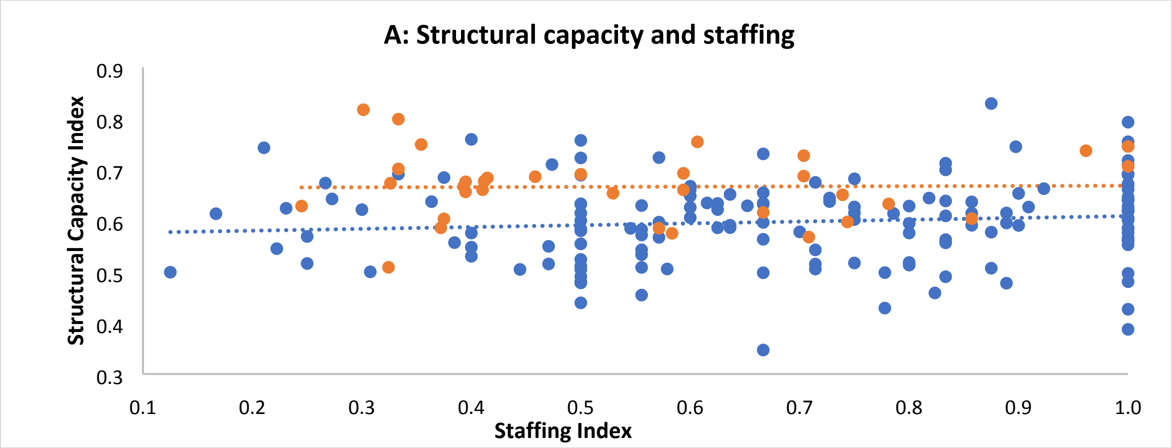

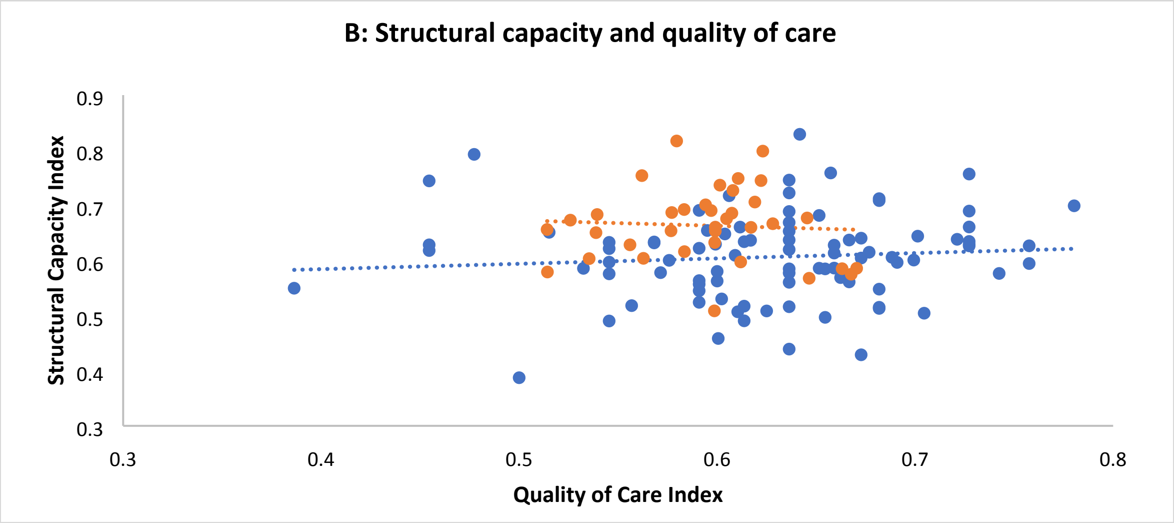

For peer review only - http://bmjopen.bmj.com/site/about/guidelines.xhtml

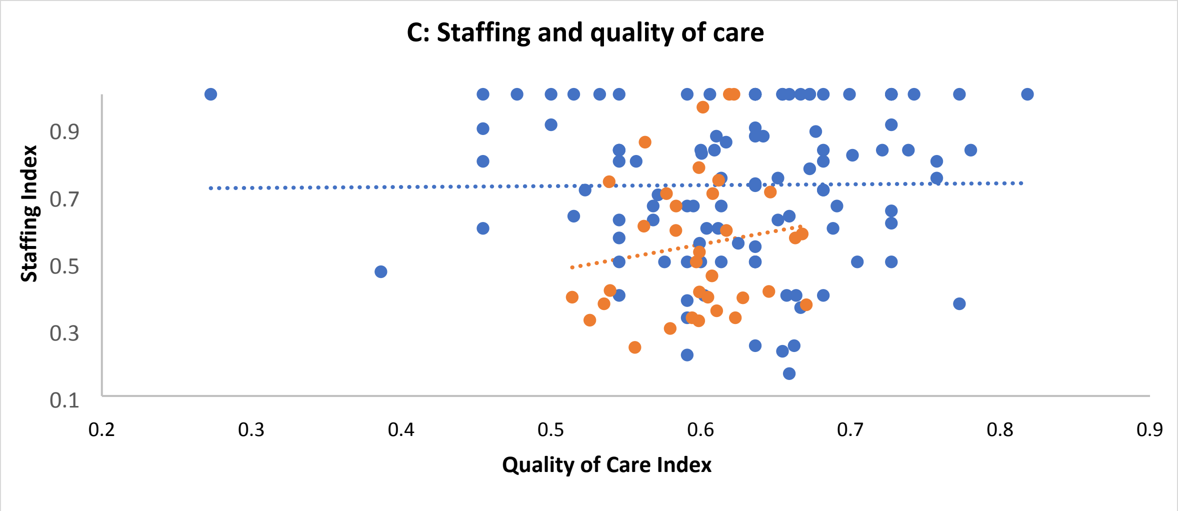

Each point represents the indexes for each facility (PHC or DH). The trend line shows the relationship between the staffing and quality of care across the PHCs (blue) and DHs (orange). Note that axis scales vary.

DISCUSSION

This study provides evidence from all DHs and a large representative sample of block PHCs in Bihar, describing the gaps that need to be addressed to improve the provision of facility-based maternal and newborn care. Gaps in the structural capacity of facilities to provide quality care in terms of basic infrastructure, availability of equipment and supplies, and appropriate staffing were identified. These are areas that will require coordinated and dedicated efforts if much needed gains are to be made towards improved quality of facility-based maternal and neonatal care.

The results revealed that DHs on average were better in terms of staffing and structural capacity in comparison with PHCs. However, the trends within the structural capacity were very similar in both the district and block facilities with availability of supplies being the lowest amongst the components of structural capacity. It is particularly concerning that DHs are missing drugs to control blood pressure and treat haemorrhage since they are supposed to deal with women who are at risk of complications.

Maintenance of hygiene is extremely important in clinical areas such as labour rooms with patients at high risk of acquiring infections. However, assessment of infrastructure readiness revealed a low level of hygiene and sanitation practices in the facilities. The study identified lack of recommended handwashing stations in different rooms and colour coded bins in the labour room. The establishment of a system of accreditation and regular monitoring of quality of hygienic care, among other interventions, may help to ensure that the facilities have the essential equipment and infrastructure in place.

The most commonly missing equipment in the DHs and PHCs were mostly pertaining to neonatal care. Access to basic neonatal care is essential to reduce neonatal mortality because between a quarter and half of all neonatal deaths happen within 24hrs of life and 75% of neonatal deaths arise in the first week of life.[22] Pre-term birth, severe infections and asphyxia have been globally identified as the main direct causes of neonatal death. Low birth weight has also been recognized as an important cause of death.[22] Low-cost interventions including tetanus toxoid vaccination,

For peer review only - http://bmjopen.bmj.com/site/about/guidelines.xhtml

exclusive breastfeeding, kangaroo mother care for low birthweight infants, and antibiotics for neonatal infections can reduce mortality.[23] However, our study revealed that skin to skin care was only being practiced by 36% of the mothers in PHCs and 24% of the mothers in DHs across Bihar. Immediate breastfeeding practice was also reported by only 24% of the mothers in PHCs and 21% mothers DHs. These findings suggest that these facilities are not ready to provide quality neonatal care and are missing simple but vital life-saving interventions.

Availability of skilled human resources is another important aspect to provide quality maternal and newborn care. The data on sanctioned posts, as reported by the facility in charge, were found to be different from those recommended by IPHS guidelines. This gap between the guidelines and actual sanctioned posts reflects the lack of translation of policies into practice. In PHCs, while the essential requirement for MOs was fulfilled in all facilities, the filled to sanctioned ratio was only 68%, indicating the need for more MOs in these facilities. In DHs, the IPHS requirement of staff nurse/ANM was fulfilled in only 15% of the facilities. In addition, lack of staff was reported as the main reason for the unavailability of services. Our results specifically indicated a lack of counsellors at both the block and district facilities. This may have contributed to less than 30% mothers reporting having received advice about their and their baby's health before discharge. The positive relationship found between the staffing and quality of care (as reported by mothers) at the DHs affirms the need to address the gaps in staffing to provide better quality of care.

Both DHs and PHCs are particularly important platforms under the health system, with DHs being the secondary referral level responsible for providing basic specialty services and PHCs being the first point of contact to a qualified doctor in the public health sector in rural areas. Given that the PHCs are not equipped to manage complicated cases, including caesarean sections or provide facilities of SNCU, it is important to have a well-functioning transport system for referrals. Our findings revealed that 34% of the PHCs did not have an emergency transport for referrals. While the Government of India recommends the provision of referral system at the facilities, no systematic step has been taken in this direction so far.[21,24] Lack of skilled staff, inadequate infrastructure and lack of accountability have been recognized as some of the key reasons for the failure of referral systems in India.[25]

This study has both strengths and limitations. The study draws on data from a large number of facilities, covering all DHs and a large representative sample of PHCs in Bihar. To our knowledge, no study of this scale has previously been conducted on facility readiness for maternal and newborn care in Bihar. The findings of this study are, however, based on data collected from a single visit to these facilities; the availability of different equipment and supplies might vary over time. The number of responses varied across and within the components of infrastructure, supplies, equipment and staffing, leading to incomplete data in some facilities. With respect to household data, women providing information on quality of care were not representative of those delivering at facilities and the sampling was not proportional to the number of deliveries at each facility. Hence, findings on quality of care at facilities as reported in the household survey should be treated as exploratory findings only. The scope of this study is limited to assessing the structural capacity of the facilities to deliver quality care and the care as reported by the mothers. However, there could be multiple other components that influence quality of care (e.g. skills and competencies of health personnel delivering care) that were not explored in this study.

CONCLUSION

For peer review only - http://bmjopen.bmj.com/site/about/guidelines.xhtml

Presence of well-functioning facilities, with required structural and staffing capacity, is crucial for providing maternal and newborn care that translates to better maternal and child outcome. Being a highly populated state with poor health outcomes, the state of Bihar requires particular attention if India is to achieve the sustainable development goals targets for maternal and newborn health. This study provides a description of the current capacity of public facilities in Bihar to provide quality maternal and neonatal care, unearthing particular gaps in neonatal equipment, infrastructure required to maintain hygiene, and staffing capacity at the facilities. Our results suggest presence of heterogeneity in the strengths and weaknesses across the facilities. A better understanding is needed to assess the cause of this variation which could help design tailored and appropriate interventions at these facilities to improve quality of care. This study lays the foundation for ongoing studies in Bihar to explore the relationship between quality of care and health outcomes. Increased focus on effective coverage and quality of facility-based care for mothers and newborns is needed if necessary gains are going to be made in saving lives in this high-burden setting.

Acknowledgements

The authors thank Sneha Lamba, Rakesh Chandra, Madhavi Misra, Tanmay Mahapatra and the entire field Team of OPM for their contribution to this manuscript.

Contributor ship Statement

T.NL. and S.F. conceived the idea for the manuscript. J.K. managed the data collection and supervised the data cleaning. J.K. led the data analysis and wrote the manuscript with the support of G.M. J.K. and G.M. jointly interpreted the data. G.M. supervised and edited the manuscript. Both T.NL. and S.F. reviewed the paper. All authors were involved in the interpretation of data, critically reviewed the manuscript and approved the final version.

Competing Interests

None.

Funding

This study was supported by Bill and Melinda Gates Foundation.

Data sharing Statement

Data used for this manuscript will be made available on request.

For peer review only - http://bmjopen.bmj.com/site/about/guidelines.xhtml

References

1. WHO, UNICEF, UNFPA, World Bank Group and UNPD. Maternal mortality in 1990-2015. 2015.

2. Sustainable Development Knowledge Platform. https://sustainabledevelopment.un.org/sdg3

3. Government of India Planning Commission. Press note on poverty estimates, 2011-12. Press Information Bureau. 2013.

4. GoI. Sample Registration System Statistical Report 2016. 2017.

5. GoI. Sample Registration System Statistical Report 2012 . New Delhi; 2013. http://www.censusindia.gov.in/vital_statistics/SRS_Reports_2012.html

6. National Family Health Survey - 4. 2015;

7. International Institute for Population Sciences. State Fact Sheet Bihar. National Family Health Survey-4. 2015. http://rchiips.org/NFHS/pdf/NFHS4/BR_FactSheet.pdf

8. Bhutta ZA, Das JK, Bahl R, Lawn JE, Salam RA, Paul VK, et al. Can available interventions end preventable deaths in mothers, newborn babies, and stillbirths, and at what cost? Lancet. 2014;384(9940):347–70.

9. Lim SS, Dandona L, Hoisington JA, James SL, Hogan MC, Gakidou E. India's Janani Suraksha Yojana, a conditional cash transfer programme to increase births in health facilities: an impact evaluation. Lancet. 2010;375(9730):2009–23.

10. Das A, Rao D, Hagopian A. India's Janani Suraksha Yojana: Further review needed. Vol. 377, The Lancet. 2011. p. 295–6.

11. Gita G, Bharti S, Shashi K, Arvind K. Trends in maternal mortality and impact of janani suraksha yojana (JSY) on maternal mortality ratio in a tertiary referral hospital. J Obstet Gynecol India. 2012;62(3):307–11.

12. Dickson KE, Simen-Kapeu A, Kinney M V., Huicho L, Vesel L, Lackritz E, et al. Every Newborn: Health-systems bottlenecks and strategies to accelerate scale-up in countries. Vol. 384, The Lancet. 2014. p. 438–54.

13. Moxon SG, Lawn JE, Dickson KE, Simen-Kapeu A, Gupta G, Deorari A, et al. Inpatient care of small and sick newborns: A multi-country analysis of health system bottlenecks and potential solutions. BMC Pregnancy Childbirth. 2015;15.

14. Sharma G, Mathai M, Dickson KE, Weeks A, Hofmeyr GJ, Lavender T, et al. Quality care during labour and birth : a multi- country analysis of health system bottlenecks and potential solutions. BMC Pregnancy Childbirth. 2015;15(Suppl 2):S2. http://www.biomedcentral.com/1471-2393/15/S2/S2

15. WHO. Standards for Improving Quality of Maternal and Newborn Care in Health Facilities. Who. 2016. http://apps.who.int/iris/bitstream/10665/249155/1/9789241511216-

For peer review only - http://bmjopen.bmj.com/site/about/guidelines.xhtml

eng.pdf?ua=1

16. Ministry of Health and Family Welfare, GoI. Rural Health Statistics, 2014-15. 2015.

17. CARE. Bihar Technical Support Programme - CARE India. 2017. https://www.careindia.org/project/technical-assistance-to-the-government-of-bihar-improving-health-nutrition-coverage-and-outcomes/

18. Das A, Chatterjee R, Karthick M, Mahapatra T, Chaudhuri I. The influence of seasonality and community-based health worker provided counselling on exclusive breastfeeding - Findings from a cross-sectional survey in India. Vol. 11, PLoS ONE. 2016.

19. WHO. Service Availability and Readiness Assessment (SARA) [Internet]. World Health Organisation (WHO). 2016. Available from: http://www.who.int/healthinfo/systems/sara_indicators_questionnaire/en/

20. Services DG of H, India M of H& FWG of. Indian Public Health Standards (Iphs) Guidelines for Primary Health Centres Revised 2012. Directorate General of Health Services Ministry of Health & Family Welfare Government of India. 2012. http://health.bih.nic.in/Docs/Guidelines/Guidelines-PHC-2012.pdf

21. Ministry of Health & Family Welfare. Indian Public Health Standards (IPHS) Guidelines for District Hospitals (101 to 500 Bedded. 2012. http://nrhm.gov.in/images/pdf/guidelines/iphs/iphs-revised-guidlines-2012/district-hospital.pdf

22. Lawn JE, Cousens S, Zupan J. 4 Million neonatal deaths: When? Where? Why? Vol. 365, Lancet. 2005. p. 891–900.

23. Martines J, Paul VK, Bhutta ZA, Koblinsky M, Soucat A, Walker N, et al. Neonatal survival: A call for action. Vol. 365, Lancet. 2005. p. 1189–97.

24. Vora KS, Mavalankar D V., Ramani K V., Upadhyaya M, Sharma B, Iyengar S, et al. Maternal health situation in India: A case study. J Heal Popul Nutr. 2009;27(2):184–201.

25. Anil Kumar Gupta, Shweta Talati, Sudip Bhattacharya AS. Health System Strengthening- Focussing on Referrals: An Analysis from India. JOJ Nurs Heal care. 2017;2(4).

For peer review only

For peer review only - http://bmjopen.bmj.com/site/about/guidelines.xhtml

APPENDIX 1

Additional methods

Infrastructure scoring

A score out of 1 was generated for each of these components. These scores were added, giving equal weight to each component and divided by the total components to generate an overall score ranging from 0 to 1, which was then converted to a percentage by multiplying by 100.

To compute a score for the component 'power/electricity' in the facilities, a value 1 was assigned to the facilities with continuous power, 0.75 to the facilities which reported occasional disruption to power in summer only, 0.5 to the facilities that were facing occasional disruption to power throughout the year, 0.25 to facilities with regular interruption to power and 0 to the facilities that reported no power supply. In cases where back-up was available that met the entire needs of the facility the above score was replaced by 1. For the 'water' score, 0.5 was assigned for availability of 24*7 running water and 0.5 if filtered water was available to patients. For telephone connection, a value 1 was assigned if it was available, else 0.

The transport score was computed by giving a score 0.5 to availability of at least one functional ambulance and an additional 0.5 to 24*7 availability. For toilets, 0.5 was assigned in case a functional toilet was available and 0.25 in case a toilet was available but not functional. Another 0.5 was assigned if the condition of the toilet was good and 0.25 if it needed repair/maintenance. For handwashing stations, 1 was assigned for the availability for elbow tap with running water and 0.5 was assigned for tap (without elbow) with running water. For computer and internet, 0.5 was assigned if the hospital had provided a computer and an additional 0.5 if there was internet connection available at the facility. For blood bank, 1 was assigned if there was a blood bank provision at the hospital, else 0.

For room availability, a score of 1 was assigned for the availability of each of the 16 rooms and then, it was added across rooms and divided by 16 to get a score out of 1. The 16 rooms were waiting room, labour room, maternity ward, immunization room, counselling room, operation theatre, store room, paediatrics, labour room eclampsia, SNCU (sick new born care unit), NBSU (new born stabilization units), septic labour room, antenatal ward, postnatal ward, postpartum ward and post-operative ward.

Equipment scoring

To compute the equipment score, a value 1 was assigned to each equipment if it was available and functional, else it was assigned 0. After computing the score for each equipment, we divided it by 48 (total equipment) to get a score out of 1 and multiplied by 100 to get the percentage distribution.

Supplies/drugs scoring

The availability of a drug was assigned a value 1, else it was assigned 0. The scores for each of the drugs were added, divided by the total (76) and then multiplied by 100 to get the percentage distribution.

For peer review only

Table S1: Infrastructure assessed

Infrastructure Components	Facility Level
Handwashing in immunization Room	PHCs, DHs
Handwashing in laboratory	PHCs, DHs
Handwashing in operation theatre	PHCs, DHs
Toilet in labour room	PHCs, DHs
Handwashing in labour room	PHCs, DHs
Transport	PHCs, DHs
Water	PHCs, DHs
Power	PHCs, DHs
Phone	PHCs, DHs
Room availability	DHs
Blood bank	DHs
Computer and internet	DHs

Abbreviations: PHC: Primary health centre, DH: District hospital

Table S2: Equipment assessed in district hospitals and primary health centres

Equipment
Caserean kit
Neonatal stethoscope
MTP suction
Light examination
NSV kit
Infantometer
Baby incubator
MTP kit
Nebulizer
Feeding tube
Shadowless lamp (OT)
Conventional vasectomy kit
Oxygen cylinder (LR)
Minilap kit
Resuscitation kit
Spotlight (OT)
Autoclave (NBCC)
Stethoscope (OT)
IV stand (OT)
Step up stool
Immunization table
Oxygen cylinder (OT)
Hub cutter (immunization room)
Partograph
Phototherapy unit

Cord clamp
Thermometer (LR)
IV cannula
Hub cutter (NBCC)
PPIUCD kit
Pump suction
Autoclave (OT)
Freeze tags
Stethoscope (LR)
Sterile gloves
Radiant warmer
Thermometer (immunization room)
Delivery kit
IUCD kit
Refrigerator
Ambu bag (NBCC)
Deep freezer
Operating table
Vaccine Carrier
BP Instrument
Labour table
Baby weighing machine
Ice packs

Information in parantheses represent the room in which the equipment was checked.

Abbreviations: MTP: Medical termination of pregnancy; NSV: Non-scalpel vasectomy; OT: Operation theatre; LR: labour room; NBCC: Newborn care corner; PPIUCD: Postpartum Intrauterine Contraceptive Device; IUCD: Intra-Uterine Contraceptive Devices; BP: Blood pressure

Table S3: Drugs assessed in district hospitals and primary health centres

Drugs
Amitriptyline
Captopril
Suxamethonium bromide
Mephenteramine
Carboprost
Miltefosine
Syrup salbutamol
Hydralazine
Omeprazole
Chlorine solution
Halothane (inhalation)
Menadione (Vit K3)
IUCD 375
Methyldopa
Simvastatin
Amphotericin B
Liposomal amphotericin B
Phytonadione
Glibenclamide
Skin disinfectant
Vitamin K
Thiopental (powder)
Dopamine
OPV
Deriphylline
BCG
Hepatitis B
DPT
Pentavalent
Frusemide
Measles
Epinephrine
Syrup vitamin A
Iron folic acid – small
Atenolol
Pentazocin chloride
Ceftriaxone
Methylegometrine (Methargine)
Nifedepine
Xylocard
Promethazine
Betamethasone
Hydrocortisone succinate

Antiseptic solution
Tetanus Toxoid
Pheneramine maleate
Adrenaline
Co-trimoxazole
Injection ranitidine
Zinc tablets
Syrup paracetamol
IUCD 380A
DEC (Diethyl Carbamazine)
Iron folic acid – Large
Amoxicillin
Metoclopramide
OCPs (Mala-N)
Ampicillin
Calcium gluconate
ECPs
Lignocaine hydrochloride 2%
Misoprostol
Condoms (Nirodh)
Ciprofloxacin
Diclofenac
Magnesium sulphate
Gentamycin
Atropine
Oxytocin
Albendazole
Ketamine
Diazepam
ORS
Dexamethasone
Metronidazole
Paracetamol

Abbreviation: IUCD: Intra-Uterine Contraceptive Devices; BCG: Bacille Calmette Guerin; DPT: Diphtheria, Pertussis and tetanus; OPV: Oral Polio Vaccine; ORS: Oral Rehydration Solution; OCP: Oral contraceptive pill; ECP: Emergency contraceptive pill

Table S4: Essential and Desirable Staff Guidelines at district hospitals (DHs) and primary health centres (PHCs) as per IPHS Guidelines

A: PHCs

IPHS Guidelines	Type A		Type B	
Staff	Essential	Desirable	Essential	Desirable
Medical Officer-MBBS	1		1	1
Medical Officer-AYUSH		1		1
Pharmacist	1		1	
Nurse-midwife (ANMs)	3	1	4	1
Laboratory Technician	1		1	

Type A PHC: PHC with delivery load of less than 20 deliveries in a month and Type B PHC: PHC with delivery load of 20 or more deliveries in a month

B: DHs

IPHS Requirements	100 Beds	200 Beds	300 Beds	400 Beds	500 Beds
Medical officer	11	13	15	19	23
Staff Nurse/ANMs	45	90	135	180	225
Lab Technician	6	9	12	15	18
Compounder/Pharmacist	5	7	9	11	13
Store Keeper	1	1	2	2	2

Table S5: Number of respondents at district hospitals (DHs) and primary health centres (PHCs).

No. of respondents at DHs	No. of respondents at PHCs	Information covered
36	190	Overall Infrastructure, Staffing
35	171	Laboratory: Infrastructure
36	179	Labour room: Infrastructure, Equipment, Supplies
35	181	Immunization room: Infrastructure, Equipment
36	166	Operation Theatre: Infrastructure, Equipment
35	177	Drug Store room: Supplies

Note: This table shows the number of respondents that were available for each of these components. The information on sub-components could vary based on the knowledge of the respondent.

Table S6: Reasons for unavailability of services at district hospitals (DHs) and primary health centres (PHCs)

	% Unavailable		*Reasons for unavailability*							
			Infrastructure		Equipment		Drugs		Supplies	
Services	DHs	PHCs	DHs	PHCs	DHs	PHCs	DHs	PHCs	DHs	PHCs
MTP	28%	81%	3%	23%	6%	37%	0%	17%	28%	71%
IUCD insertion	0%	3%	0%	1%	0%	1%	0%	0%	0%	2%
IUCD removal	0%	3%	0%	1%	0%	1%	0%	0%	0%	2%
Post-partum IUCD insertion	0%	16%	0%	6%	0%	7%	0%	3%	0%	13%
Non-scalpel vasectomy	11%	70%	3%	16%	6%	31%	3%	10%	6%	61%

For peer review only

Conventional vasectomy	19%	42%	3%	10%	3%	15%	0%	2%	6%	35%
Minilap tubectomy	3%	8%	3%	2%	0%	2%	0%	0%	3%	7%
Post-partum minilap sterilization	3%	28%	0%	7%	3%	12%	0%	4%	3%	22%
Laparoscopic sterilization	92%	95%	19%	23%	47%	50%	3%	13%	61%	81%
Distribution of condoms	6%	3%	0%	0%	0%	0%	3%	1%	0%	2%
Distribution of OCPs	3%	3%	0%	0%	0%	0%	3%	1%	0%	2%
Distribution of ECPs	11%	6%	0%	1%	0%	0%	11%	2%	0%	3%
Normal delivery	0%	5%	0%	2%	0%	2%	0%	1%	0%	3%
Caesarian section	3%	-	3%	-	3%	-	0%	-	0%	-
Managing complicated pregnancies	0%	31%	0%	10%	0%	17%	0%	5%	0%	24%
Managing delivery cases with eclampsia	8%	20%	0%	4%	0%	10%	0%	5%	8%	14%
Registration of pregnancies	0%	2%	0%	0%	0%	1%	0%	0%	0%	1%
Antenatal care visits	0%	2%	0%	0%	0%	1%	0%	0%	0%	1%
Distribution of IFA tablets	0%	5%	0%	0%	0%	1%	0%	4%	0%	1%
Tetanus toxoid vaccinations	3%	1%	3%	0%	0%	1%	0%	0%	0%	1%
VDRL tests during antenatal visits	17%	30%	3%	6%	11%	17%	3%	8%	3%	16%
BP tests during antenatal visits	0%	1%	0%	0%	0%	1%	0%	0%	0%	1%
HB tests during antenatal visits	0%	8%	0%	1%	0%	5%	0%	4%	0%	5%
Weighing of newborns at birth	0%	4%	0%	2%	0%	2%	0%	1%	0%	3%
Neonatal resuscitation in cases with asphyxia	3%	7%	3%	3%	0%	5%	0%	2%	3%	6%
Post-partum check ups within 48hrs of delivery	0%	5%	0%	2%	0%	2%	0%	1%	0%	4%
Post-partum check ups b/w 48hrs &14 days of delivery	0%	11%	0%	3%	0%	3%	0%	1%	0%	6%
Immunization sessions	53%	2%	3%	0%	6%	1%	6%	0%	6%	2%
Treatment for diarrhea	0%	3%	0%	1%	0%	2%	0%	1%	0%	2%
Treatment for acute respiratory infections	8%	23%	3%	7%	3%	8%	0%	5%	3%	20%

Abbreviations: MTP: Medical termination of pregnancy; IUCD: Intra-Uterine Contraceptive Devices; OCP: Oral contraceptive pill; ECP: Emergency contraceptive pill; IFA: Iron folic acid

For peer review only

Table S7: Most commonly missing and available structural capacity items across primary health centres and district hospitals

	Infrastructure		Equipment		Drugs	
	**Most commonly missing**	**Most commonly available**	**Most commonly missing**	**Most commonly available**	**Most commonly missing**	**Most commonly available**
**Primary health centres**	Handwashing in immunization room (13%)	Water (81%)	Neonatal stethoscope (16%)	Deep freezer, Ambu bag (97%)	Amitriptyline (12%)	ORS (95%)
	Handwashing in laboratory (37%)	Power (87%)	MTP suction (17%)	Operating table, labour table, BP Instrument, vaccine carrier (98%)	Captopril (14%)	Dexamethasone (99%)
	Handwashing in OT (60%)	Phone (93%)	Light examination (20%)	Baby weighing machine, ice packs (99%)	Suxamethonium bromide, mephenteramine (16%)	Paracetamol, metronidazole (100%)
**District hospitals**	Handwashing in immunization room (19%)	Water (97%)	Infantometer, nebulizer, baby incubator (14%)	IV cannula, PPIUCD kit, ice packs (94%)	Captopril, phytanodiane, simvastin, IUCD 35 (3%)	Atropine, DPT (94%)
	Handwashing in laboratory (47%)	Power (99%)	Neonatal stethoscope, MTP suction (39%)	Vaccine carrier, operating table, delivery kit, oxygen cylinder (97%)	Amritriptyline (6%)	Dexamethasone, metronidazole, paracetamol, BCG, OPV, measles, pentavalent (97%)
	Room Availability (64%)	Transport (100%)	Light examination, resuscitation kit, phototherapy unit (42%)	Labour table, MTP kit, IUCD kit, Ambu bag, IV stand (100%)	Glibenclamide, suxamethonium bromide, syrup vit A (8%)	Diazepam, tetanus toxoid (100%)

For peer review only

STROBE Statement—Checklist of items that should be included in reports of ***cross-sectional studies***

	Item No	Recommendation	Page No
**Title and abstract**	1	(*a*) Indicate the study's design with a commonly used term in the title or the abstract	1
		(*b*) Provide in the abstract an informative and balanced summary of what was done and what was found	1
**Introduction**			
Background/rationale	2	Explain the scientific background and rationale for the investigation being reported	2
Objectives	3	State specific objectives, including any prespecified hypotheses	3
**Methods**			
Study design	4	Present key elements of study design early in the paper	3
Setting	5	Describe the setting, locations, and relevant dates, including periods of recruitment, exposure, follow-up, and data collection	3
Participants	6	(*a*) Give the eligibility criteria, and the sources and methods of selection of participants	4
Variables	7	Clearly define all outcomes, exposures, predictors, potential confounders, and effect modifiers. Give diagnostic criteria, if applicable	5
Data sources/ measurement	8*	For each variable of interest, give sources of data and details of methods of assessment (measurement). Describe comparability of assessment methods if there is more than one group	*4*
Bias	9	Describe any efforts to address potential sources of bias	2
Study size	10	Explain how the study size was arrived at	3
Quantitative variables	11	Explain how quantitative variables were handled in the analyses. If applicable, describe which groupings were chosen and why	4
Statistical methods	12	(*a*) Describe all statistical methods, including those used to control for confounding	5,6
		(*b*) Describe any methods used to examine subgroups and interactions	6
		(*c*) Explain how missing data were addressed	6
		(*d*) If applicable, describe analytical methods taking account of sampling strategy	
		(*e*) Describe any sensitivity analyses	
**Results**			
Participants	13*	(a) Report numbers of individuals at each stage of study—eg numbers potentially eligible, examined for eligibility, confirmed eligible, included in the study, completing follow-up, and analysed	6
		(b) Give reasons for non-participation at each stage	6
		(c) Consider use of a flow diagram	
Descriptive data	14*	(a) Give characteristics of study participants (eg demographic, clinical, social) and information on exposures and potential confounders	6,7
		(b) Indicate number of participants with missing data for each variable of interest	6
Outcome data	15*	Report numbers of outcome events or summary measures	6
Main results	16	(*a*) Give unadjusted estimates and, if applicable, confounder-adjusted estimates and their precision (eg, 95% confidence interval). Make clear which confounders were adjusted for and why they were included	7-12

For peer review only - http://bmjopen.bmj.com/site/about/guidelines.xhtml

		(*b*) Report category boundaries when continuous variables were categorized	
		(*c*) If relevant, consider translating estimates of relative risk into absolute risk for a meaningful time period	
Other analyses	17	Report other analyses done—eg analyses of subgroups and interactions, and sensitivity analyses	7-12
**Discussion**			
Key results	18	Summarise key results with reference to study objectives	13,14
Limitations	19	Discuss limitations of the study, taking into account sources of potential bias or imprecision. Discuss both direction and magnitude of any potential bias	14
Interpretation	20	Give a cautious overall interpretation of results considering objectives, limitations, multiplicity of analyses, results from similar studies, and other relevant evidence	13,14
Generalisability	21	Discuss the generalisability (external validity) of the study results	14
**Other information**			
Funding	22	Give the source of funding and the role of the funders for the present study and, if applicable, for the original study on which the present article is based	15

*Give information separately for exposed and unexposed groups.

Note: An Explanation and Elaboration article discusses each checklist item and gives methodological background and published examples of transparent reporting. The STROBE checklist is best used in conjunction with this article (freely available on the Web sites of PLoS Medicine at http://www.plosmedicine.org/, Annals of Internal Medicine at http://www.annals.org/, and Epidemiology at http://www.epidem.com/). Information on the STROBE Initiative is available at www.strobe-statement.org.

For peer review only

BMJ Open

Readiness of public health facilities to provide quality maternal and newborn care across the state of Bihar, India: a cross-sectional study of district hospitals and primary health centres

Journal:	*BMJ Open*
Manuscript ID	bmjopen-2018-028370.R1
Article Type:	Research
Date Submitted by the Author:	17-Apr-2019
Complete List of Authors:	kaur, Japneet; Oxford Policy Management,<br>Franzen, Samuel; Oxford Policy Management, ; University of Oxford, Centre for Tropical Medicine and Global Health<br>Newton-Lewis, Tom; Oxford Policy Management<br>Murphy, Georgina ; Kenya Medical Research Institute/Wellcome Trust Research Programme, Health Services Unit; Bill and Melinda Gates Foundation
**Primary Subject Heading**:	Health services research
Secondary Subject Heading:	Health economics, Health policy
Keywords:	Health & safety < HEALTH SERVICES ADMINISTRATION & MANAGEMENT, Health economics < HEALTH SERVICES ADMINISTRATION & MANAGEMENT, Health policy < HEALTH SERVICES ADMINISTRATION & MANAGEMENT, Quality in health care < HEALTH SERVICES ADMINISTRATION & MANAGEMENT

SCHOLARONE™
Manuscripts

Full Title: Readiness of public health facilities to provide quality maternal and newborn care across the state of Bihar, India: a cross-sectional study of district hospitals and primary health centres

Short title: Readiness of health facilities to provide quality maternal and newborn care in Bihar, India

Authors: Japneet Kaur* [1], Sam Frazen[2], Tom Newton-Lewis[1], Georgina A. V. Murphy[1,3]

Affiliations:

1.Oxford Policy Management(OPM), Delhi, India

2. Oxford Policy Management(OPM), Oxford, UK

3. Centre for Tropical Medicine and Global Health, University of Oxford, UK

***Corresponding author:** Japneet Kaur, Oxford Policy Management, 4/6 First Floor, Siri Fort Institutional Area, New Delhi, India Email: japneetkaur58@gmail.com. Tel: (+91) 011-48081111

Key words: neonatal care, maternal care, newborn, infrastructure, newborn mortality, maternal mortality, district hospital, primary health care, audit, staffing, quality of care, Bihar, India

Abstract

Introduction: Poor access to quality healthcare is one of the most important reasons of high maternal and neonatal mortality in India, particularly in poorer states like Bihar. India has implemented initiatives to promote institutional maternal deliveries. It is important to ensure that health facilities are adequately equipped and staffed to provide quality care for mothers and newborns.

Methods: We conducted a cross-sectional study of 190 primary health centres (PHCs) and 36 district hospitals (DHs) across all districts in Bihar to assess the readiness of facilities to provide quality maternal and neonatal care. Infrastructure, equipment and supplies, and staffing were assessed using the WHO service availability and readiness assessment and Indian public health standard (IPHS) guidelines. Additionally, we used household survey data to assess the quality of care reported by mothers delivering at study facilities.

Results: PHCs and DHs were found to have 61% and 67% of the mandated structural components to provide maternal and neonatal care, on average, respectively. DHs were, on average, slightly better equipped in terms of infrastructure, equipment and supplies by comparison to PHCs. DHs were found to be inadequately prepared to provide neonatal care. Lack of recommended handwashing stations and bins at both DHs and PHCs suggested low levels of hygiene. Only half of the essential drugs were available in both DHs and PHCs. While no association was revealed between structural capacity and patient-reported quality of care, adequacy of staffing was positively associated with the quality of care in DHs.

Conclusion: Examining all DHs and a representative sample of PHCs in Bihar, this study revealed the gaps in structural components that need to be filled to provide quality care to mothers and

For peer review only - http://bmjopen.bmj.com/site/about/guidelines.xhtml

newborns. Access to quality care is essential if progress in reducing maternal and neonatal mortality is to be achieved in this high-burden state.

Strengths and limitations of this study

- The findings of this study are based on data collected from a single visit to these facilities; the availability of different equipment and supplies might vary over time.
- There is incomplete data in some facilities. Therefore, the number of responses varied across and within the components of infrastructure, supplies, equipment and staffing.
- With respect to household data, women providing information on quality of care were not representative of those delivering at facilities and the sampling was not proportional to the number of deliveries at each facility
- This study is limited to assessing the structural capacity of the facilities to deliver quality care as reported by the mothers. However, there could be multiple other components that influence quality of care (e.g. skills and competencies of health personnel delivering care) that were not explored in this study.
- The study covers all DHs and a large representative sample of PHCs in Bihar. To our knowledge, no study of this scale has previously been conducted on facility readiness for maternal and newborn care in Bihar.

INTRODUCTION

Progress has been made in reducing maternal and newborn mortality in India over the last three decades. Between 1990 and 2015, maternal mortality reduced from 556 to 174 per 100,000 live births and neonatal mortality reduced from 54 to 29 per 1,000 live births.[1–3] However, considerable further improvements will be needed if India is to reach the Sustainable Development Goal of reducing maternal mortality to less than 70 per 100,000 births and neonatal mortality to at least as low as 12 per 1,000 live births by 2030.[4]

These goals will be particularly challenging for Bihar, the third most populated state in India (approximately 104 million). Bihar struggles with persistent poverty (34% of the population lives below the poverty line) and poor health outcomes (neonatal mortality rate of 27 per 1,000 live births and maternal mortality rate of 208 per 100,000 live births).[5–7] Only 63% of the pregnant women deliver in a health facility in Bihar, which is 12% lower than the national average.[8,9] This is an important area that needs attention since the biggest gains in survival are estimated to be achieved through facility-based maternal care provided at the time of childbirth and the immediate postpartum period along with newborn care.[10]

In recognition of the importance of facility-based maternal and newborn care, India has implemented many initiatives to encourage institutional deliveries. The most ambitious of these is the Janani Suraksha Yojana (JSY) programme offering conditional cash transfers to women of low socio-economic status for delivering at a health facility.[2] Despite the success of JSY in increasing institutional deliveries, provision of quality care has been highlighted as an important barrier for the programme to have the intended effect on health outcomes.[11,12] Addressing the gaps in facility readiness has been considered as an important factor in improving quality of

For peer review only - http://bmjopen.bmj.com/site/about/guidelines.xhtml

care.[13] Poor availability of clinical services due to lack of infrastructure has been recognized as one of the most common bottleneck to providing essential maternal and new born services in India.[14] Rammohan et al. 2013 report lack of transport facilities for pregnant women as one of the major bottlenecks to access emergency obstetric care in India.[15] Capacity in terms of equipment and staff availability needs to be built to detect and manage obstetric emergencies.[16]

It is crucial to ensure that facilities are adequately resourced and equipped to deliver essential maternal and newborn care.[14,17–19] The role of quality factors such as infrastructure, equipment, supplies and staffing is acknowledged, but little research has been done to quantify and describe these gaps in detail. This is needed if interventions to strengthen quality are to be appropriately designed and targeted to be effective.[20]

The public health system in India comprises of a three-tier system, namely, primary care at the village level, secondary care at the sub-district and district levels and tertiary levels of health care at the regional level. The district hospital (DH) is an essential medium of secondary level of health care with an objective to provide curative, preventive and promotive health care services to the people in the district. Linked to every DH are health centres providing primary care, including sub-divisional hospitals, community health centres (CHCs), primary health centres (PHCs) and sub-centres. PHCs are crucial to the health system as they form the first point of contact to a qualified doctor of the public sector for the patients. There are two kinds of PHCs, one is called additional PHC which mainly does clinical work and the other is block PHC which also exercises administrative powers in the entire block. Serving a population between 20,000 and 30,000, PHCs act as a referral unit for six sub-centres and refer out cases to CHCs and higher order facilities. In Bihar, there are 36 DHs, 70 CHCs, 9729 sub-centres and 1883 PHCs (including 534 block PHCs).[21]

This study aims to (a) assess and highlight structural and staffing gaps in the public health facilities, specifically, PHCs and DHs in Bihar, that need to be addressed, to deliver quality maternal and newborn services (b) understand the relationship between structural and process quality metrics for maternal and newborn health services. This study is based on the data collected in the baseline assessment of Bihar Technical Support Programme (BTSP). BTSP is a large multi-year programme funded by the Bill and Melinda Gates Foundation and implemented by CARE India with OPM as monitoring and evaluation partner.[22] Working closely with Government of Bihar's Departments of Health and Family Welfare and Social Welfare, CARE India's interventions aim to strengthen the health system and improve the quality of care to improve reproductive, maternal, newborn, child, adolescent and nutrition (RMNCH+N) outcomes.

METHODS

We conducted a cross-sectional study of health facilities in Bihar during July-October 2016. Facility surveys were conducted in block (sub-district) and district level government-run public health facilities. This study also uses household maternal and child health survey data collected during October-December 2016 by CARE India.

Study population and sampling

For peer review only - http://bmjopen.bmj.com/site/about/guidelines.xhtml

There are 36 district hospitals in Bihar, all of which were invited to participate in the facility survey. There are 534 blocks (sub-districts) in Bihar, 190 of which were sampled for the facility survey. The number of blocks vary widely per district. Hence, blocks were sampled proportionally according to the total number of blocks per district. The selected sample had blocks ranging from 1 to 9 per district with a median of 6 blocks. Each block contains one block PHC, all of which (from the 190 sampled blocks) were included in the facility survey.

Household survey data were collected using five different questionnaires for mothers who had a child belonging to the following five age groups: i) 0-2; ii) 3-5; iii) 6-8; iv) 9-11; and v) 12-23 months old. A mixed sampling methodology of population based-estimation and lot quality assurance sampling (LQAS) (a small sample survey design based on binomial distribution) was used [23]. The sampling 'lots' in this survey were the blocks/sub-districts. All 534 blocks in 38 districts were included in the study data collection. The number of Anganwadi Centers (AWC, village level institutions providing basic health care services) sampled from each block was determined using proportional allocation, however if this resulted in a sample of less than 19 AWCs, then 19 AWCs were sampled in order to meet a minimum sample threshold per block. The sampled AWC were selected within each block using simple random sampling. Five households per AWC were selected, with one each from mother of following five age groups- (a) 0-2 (b) 3-5 (c) 6-8 (d) 9-11 (e) 12-23. In total, 15667 AWCs were selected ranging from 19 to 123 per block.

Within each sampled AWC catchment area, households were identified through systematic sampling.[23] Briefly, an index household was chosen within each AWC catchment area using a random number table. Starting with the index household, data collectors visited every fifth household looking for eligible mothers. This approach aimed to obtain a wide distribution of households (minimizing the effect of clustering), while remaining feasible and practical for data collection purposes. The pilot phase of the study did not observe any significant differences in household characteristics when alternative sample intervals of 10th, 15th, and 20th households were selected. The data collectors continued moving in a circular manner, following the 'right-hand rule', until five eligible households had been interviewed per AWC catchment area, one household for each age group questionnaire.

To reduce the recall bias, data on quality care presented in the analysis were restricted to mothers with children aged between 0-2 months. Of the mothers who also delivered at the DHs or PHCs that were covered in the facility survey (ranging from 1 to 17 mother per facility) were included in this analysis.

Data collection

Facility survey

Data were collected using a standardised structured survey tool designed based on the Service Availability and Readiness Assessment (SARA) tool developed by the World Health Organisation (WHO) and the United States Agency for International Development.[24] The tool was modified for the Indian context using the Indian Public Health Standards (IPHS) guidelines.[25,26] To evaluate the structural capacity of the facility, the availability and condition of infrastructure, equipment, and supplies in different departments, including the labour room, New Born Care Corner (NBCC), immunization room, laboratory, operation theatre, drug store, and data operation

For peer review only - http://bmjopen.bmj.com/site/about/guidelines.xhtml

were assessed. Information on infrastructure and equipment was collected through interviews with the facility-in-charge and staff nurse as well as through direct observation. The pharmacist or drug store-in-charge was interviewed, and the responses were validated through the drug register to collect information on supplies availability.

The Medical Officer in Charge (MOIC) at the PHCs and Hospital Manager at the DHs were also interviewed to obtain information on the number of health personnel employed at the facilities and the number of personnel that were sanctioned (number of staff expected to be employed) to the facilities for each of the health cadres, including medical officers (MOs), staff nurses, auxiliary nurse midwifery(ANMs), laboratory technicians, and pharmacists. This information was also cross-checked with the facility registers.

Availability of 30 services related to family planning, safe delivery, antenatal care, and neonatal and child care was assessed and the reasons for unavailability were asked from the MOIC in PHCs and the Hospital Manager in DHs.

Three pilot tests were conducted in the facilities outside the study sample to refine the survey tool and to train the enumeration team. The survey was conducted by 60 enumerators over the four month period. Enumerators all had prior experience in conducting facility surveys and received further training over 10 days on using the study tool and conducting this survey.

Periodic data checks for completeness and outliers were conducted by a data management team in Patna, Bihar. Where information was missing due to absenteeism or lack of time provided by the respondent, a second visit to those facilities was organized.

LQAS Household survey

One-to-one interviews were conducted with consenting and eligible mothers by trained data collectors, using a standardised questionnaire and following standard operating procedures. Information collected from mothers and of interest to this study included the household characteristics, the place of delivery, and care received at the place of delivery.

Patient and Public involvement

Patients were not involved in the study.

Data Analysis

Data analysis was conducted using Stata version 13 (Stata Corporation, USA). The current status of the facilities was assessed on three broad parameters, namely, the structural capacity, staffing, and the quality of care provided at the facilities.

Structural Capacity

The structural capacity of the facilities was assessed by computing readiness scores of 0-1 for infrastructure, equipment and supplies. “Infrastructure readiness” included the availability as well as the condition of different components, wherever applicable. For equipment, “readiness” implied the availability as well as functionality of the equipment and for supplies, readiness was defined by availability.[24]

For peer review only - http://bmjopen.bmj.com/site/about/guidelines.xhtml

Infrastructure readiness of the facilities included nine broad components (such as power, water, transport, handwashing stations) at the PHCs.[24] An additional three components (availability of different rooms, computer and internet, and blood bank) were assessed for DH infrastructure score (details of components are listed in **appendix table S1**).

The equipment readiness of the facilities was assessed by scoring the availability and functionality of 48 essential (according to IPHS guidelines) maternal and newborn health equipment (items listed in **appendix table S2**). A score of 1 was assigned if the equipment was observed to be available and in a functional state. In case of unavailability or available but not functional equipment, a score of 0 was assigned. Similarly, supplies readiness was assessed by considering the availability of 76 essential maternal and child health drugs that were expected at the facilities as per the IPHS guidelines (listed in **appendix table S3**). The mean across the three components of infrastructure, equipment and supplies was computed to generate a score for structural capacity ranging from 0 to 1 per facility. The mean across facilities was computed to get an overall score for structural capacity. Detailed methods of scoring have been provided in **the appendix**.

Staffing Index

We assessed the availability of human resources by computing the ratio of filled to sanctioned positions, as reported by the MOIC and the Hospital Manager or equivalent authority in charge in the PHCs and DHs, for each health cadre in each facility. The ratio of total filled to total sanctioned positions for permanent staff, combining all cadres, was computed to generate an overall staffing index for each facility.

The availability of health staff was also compared with the essential requirements mandated by IPHS guidelines. In PHCs, we considered staff requirement based on the monthly delivery load of more than 20, as provided by the IPHS guidelines.[25] In DHs, the staff requirement based on the bed strength were rounded down to compare with the mandated guidelines.[26] For instance, for DHs with less than or equal to 200 beds, we considered the staff requirements for 100 beds as defined by IPHS guidelines. For ANMs, the IPHS requirement of 0.45 staff per bed was considered. **(appendix table S4)**.

The relationship between availability of services (that were unavailable in at least 10% of the PHCs and DHs) and structural capacity and staffing index was explored by assessing the pairwise correlation coefficients between the indices at the facility.

Quality of care

Our primary aim was to describe the structural readiness of facilities to provide essential maternal and new-born services. We also conducted analyses of household survey data to explore the quality of care at facilities as reported by women who both participated in the household survey and delivered at study facilities.

Each mother was asked 11 questions during the household survey pertaining to the treatment and care that they and their newborns received during delivery. Each question was assigned a score of 0 (not performed/don’t know) or 1 (performed). Household survey data was merged with facility data by matching the names of facilities where mothers delivered with the facility names collected during facility assessment survey. A quality of care index for each PHC and DH was

For peer review only - http://bmjopen.bmj.com/site/about/guidelines.xhtml

generated by taking the average score of the 11 questions for all those household survey participants who delivered within the facility. All data were assessed at the facility level.

The relationship between structural capacity, staffing, and quality of care indexes were visually explored using scatter plots and trend lines as part of this exploratory analysis.

Ethics and permission

Ethical approval was granted by the Indian Institutional Review Board. At each facility, the purpose of the study was explained and informed consent was obtained from the MOIC and the Hospital Manager or equivalent authority in charge in the PHCs and DHs, respectively. For the household survey, ethics approval was obtained from Ashirwad Ethics Committee, Ashirwad Hospital and Research Center, Ulhasnagar, India and informed consent was taken from the mothers

RESULTS

The number of facilities assessed for each component of structural capacity and staff availability varied (range: 35-36 DHs and 166-190 PHCs) due to missing data and depending on the availability of respondents during the time of the survey **(appendix table S5).** Household survey data were available from 671 mothers who delivered in 107 of the 190 study PHCs and 1419 mothers who delivered in across all 36 study DHs.

Facility characteristics

Most PHCs (95%) were functional for 24 hours per day, but 40% of them were not accessible throughout the year. A dedicated labour room, maternity ward, operation theatre and store room was found to be available in most PHCs (94%, 96%, 89% and 96% respectively); an immunisation room was available in only 76% of the PHCs. While the IPHS guidelines recommend each PHC to have six beds, the number of sanctioned and available beds, as reported by the MOIC, varied. Eight PHCs reported having no beds, four of which nonetheless conducted maternal deliveries.

All DHs were found to have a dedicated labour room and maternity ward, but specialised units for antenatal care and for post-natal care were available in only 69% and 56% of the DHs, respectively. As per the IPHS guidelines, every DH should have a provision for Special New Born Care units (SNCUs); however, this unit was found in only 21 of the 36 DHs (58%). In DHs, the number of beds recommended by IPHS guidelines varies between 75 to 500 depending on the size, terrain, and population of the district; however, in Bihar, we identified four DHs with fewer than 75 beds available.

Availability of services

Of the 30 services assessed in 36 DHs and 189 PHCs, seven (23%) and 12 (40%) services were unavailable in at least 10% of the facilities, respectively. Most of the commonly unavailable services were related to family planning including medical termination of pregnancy (MTP), non-scalpel vasectomy, conventional vasectomy, and laparoscopic sterilization. Venereal disease research laboratory (VDRL) tests conducted during ANC visits were unavailable in 17% and 30% of the DHs and PHCs, respectively (**figure 1).**

For peer review only - http://bmjopen.bmj.com/site/about/guidelines.xhtml

For both PHCs and DHs, the main reason for the lack of these services was reported to be lack of required human resources (**figure 1** and **appendix table S6**). In PHCs, lack of equipment was reported to be the second most important factor for the unavailability of services such as MTP, non-scalpel vasectomy and laparoscopic sterilization. Lack of equipment was also the reason for unavailability of laparoscopic sterilization in 47% of the 36 DHs.

Structural capacity

Overall, the average structural capacity across PHCs was 60% (range: 35-83%) and 66% (range: 51-82%) across DHs. DHs were slightly better equipped on average in terms of availability of infrastructure, equipment and drug supplies (78%, 70%, 53%, respectively) compared with PHCs (63%, 65%, and 50%, respectively). Results varied greatly between facilities, particularly among PHCs (**figure 2**).

Infrastructure

Infrastructure score at the DHs and PHCs varied with a range of 60-92% and 32-90%, respectively. Of the 12 items assessed in DHs, five (hand washing station in the labour room, telephone connection, water, power and transport) had an average readiness score of greater than 90%. In PHCs, telephone connection was the only component with an average readiness score of over 90% across facilities. Readiness was particularly low with respect to hand washing stations in the immunisation room and laboratory in both DHs and PHCs (**appendix table S7**).

Of all the items accessed in the labour room, the availability of different colour coded bins to segregate waste into infectious and non-infectious sources was the lowest in both PHCs and DHs (54% and 63%, respectively). Emergency transport for referrals was available in only 66% of the PHCs, whereas the DHs performed well in this regard with all DHs having emergency transport available for referrals.

Equipment and supplies

Equipment score at the DHs ranged between 54% and 92%; the corresponding range at PHCs was 40-90%. Neonatal stethoscope and MTP suction were the two most commonly missing items of equipment in PHCs; whereas, in DHs, infantometer and nebulizer were the two most commonly missing items (**appendix table S7**). In the labour room specifically, light examination, feeding tube and oxygen cylinder were the most commonly missing items of equipment in both the DHs and PHCs.

Availability of drugs was the weakest performing area of structural assessment for both DHs and PHCs (figure 1), with only half (50% and 52% on average, respectively) of the essential drugs (n=76) being available. Drug score varied with a range of 25-100% across DHs and 14-82% across PHCs. Of 25 essential drugs that should be available in the labour room as per the IPHS guidelines, 62% and 72% were available on average in PHCs and DHs, respectively. Carboprost, hydralazine and methyldopa were the least commonly available of the drugs in both the PHCs and DHs.

For peer review only - http://bmjopen.bmj.com/site/about/guidelines.xhtml

Staff availability

The overall average staffing index was 69% (range: 11-100%) in PHCs, indicating 31% of health worker sanctioned positions, as reported by the MOIC, being unfilled. The average staffing index at PHCs was found to be the highest for the ANMs, indicating a high proportion of sanctioned positions being filled (**table 1**). However, the requirement of ANMs, as mandated by the IPHS guidelines, was fulfilled in only 42% of the PHCs **(table 1)**. The mandate of having at least one MO at a PHC was fulfilled at all PHCs. However, the sanctioned positions, as reported by the MOIC, varied and the average staffing index of available to sanctioned MOs was 70% for contractual (n =129) and 68% for permanent staff. The proportion of positions filled in PHCs was the lowest for laboratory technicians (27%). These technicians were, however, supplemented by contractual workers, for whom 92% of sanctioned positions were filled. RMNCH+ counsellors were available in only four PHCs (2%) and family planning counsellors in six PHCs (3%). None of the PHCs had an infant and young child feeding counsellor.

In DHs, the overall staffing index for three cadres was 55% (range 24-100%). The staffing index amongst the health personnel in DHs was found to be similar to PHCs (**table 1**); the staffing index was also highest for ANMs (78%) and lowest (35%) for laboratory technicians in DHs. For ANMs, the IPHS requirement of 0.45 staff per bed was fulfilled in only 15% of the DHs **(table 1)**. The average staffing index for MOs was 52% and the requirement of essential MOs as per the IPHS guidelines was fulfilled in 53% of the DHs. Nearly 60% of the DHs had less than half of the sanctioned positions for MOs and nurses filled.

Table 1: Average filled/sanctioned positions for staff and IPHS requirement fulfillment for district hospitals (DHs) and primary health centres (PHCs)

Designation	Average filled to sanctioned- DHs	Avgerage filled to sanctioned- PHCs	% DHs fulfilling IPHS requirements	% PHCs fulfilling IPHS requirements
Medical officer	52% (34)	68% (190)	53% (34)	100% (190)
Staff Nurse	44% (33)	42% (48)	15% (33)*	-
Auxiliary nurse midwife	78% (24)	81% (173)		42% (173)
Laboratory technician	35% (32)	27% (148)	0% (32)	27% (148)
Compounder/pharmacist	56% (32)	63% (171)	16% (32)	70% (171)
Store keeper	58% (28)	57% (101)	61% (28)	57% (101)

*Only permanent positions are considered. Cases where information on sanctioned positions was missing were excluded. Medical officers include physicians, obstetricians, paediatricians and anaesthetists. PHC IPHS guidelines mention to appoint at least 4 Nurse- Midwives. We consider at least 4 ANMs for each facility since the information for Staff Nurse is unavailable for most facilities. *DH IPHS guidelines mention the requirement for staff nurse/ANM combined and hence we consider the combined availability of staff nurse and ANM*

For peer review only - http://bmjopen.bmj.com/site/about/guidelines.xhtml

Relationship between service availability indexes

In PHCs, all three components of structural capacity index, including infrastructure, equipment and supplies, had significantly positive correlation with the availability of the 12 services at 5% level of significance. For DHs, availability of seven services that were unavailable in at least 10% DHs, had positive correlation with equipment, supplies and staffing index at 5% level of significance.

Reported quality of care

When asked if 11 essential pre- and post-partum services had been carried out, the responses were similar between DHs and PHCs (**table 2**). Almost all mothers reported that hygiene and newborn warmth practices of wearing gloves, wiping the baby dry and wrapping the baby were being practiced in both PHCs and DHs. Provision of skin-to-skin contact was reported by fewer than half of women, regardless of facility type. Measuring blood pressure and advising mothers about their and their baby's health before discharge were received by less than 30% of the mothers.

Table 2: Quality of care reported by mothers delivering at the primary health centres (PHCs) and district hospitals (DHs)

	PHCs (n =671)			DHs (n = 1419)		
**Quality of Care**	**Yes**	**No**	**Don't Know**	**Yes**	**No**	**Don't Know**
Was the baby wrapped in a clean cloth after birth?	97.91%	1.34%	0.75%	96.41%	1.20%	2.40%
Did this person wear gloves before conducting your delivery?	96.87%	1.64%	1.49%	95.49%	1.20%	3.31%
Was the baby wiped dry after delivery?	95.68%	2.53%	1.79%	93.31%	2.47%	4.23%
Was the baby weighed after delivery?	92.55%	3.73%	3.73%	88.94%	5.64%	5.43%
After delivery, was nothing applied to the cord?	91.36%	8.67%	0%	85.27%	14.73%	0%
Did the person wash hands with soap before conducting your delivery?	76.15%	3.73%	20.12%	73.50%	5.14%	21.35%
Was the baby placed on the mother's abdomen immediately after birth?	49.78%	42.92%	7.30%	40.03%	48.98%	10.99%
Were you advised by the nurse or anyone else to keep the baby naked on your chest, next to your skin?	35.77%	63.49%	0.75%	23.82%	75.26%	0.92%
Did you breastfeed your baby immediately after delivery?	24.29%	75.71%	0%	21.17%	78.48%	0.24%
Was any advice given to you regarding your health or your baby's health before you were discharged from the facility?	29.06%	70.94%	0%	18.60%	81.40%	0%
Was blood pressure measured after delivery, before discharge?	9.99%	90.91%	0%	8.67%	91.33%	0%

For peer review only - http://bmjopen.bmj.com/site/about/guidelines.xhtml

Relationship between quality indexes

No clear relationship between the facility structural capacity index (composite score for infrastructure, equipment, and drugs), the staffing index (ratio of sanctioned to filled positions), or the quality of care index (average score for 11 facility-based care services among women per facility) was found for PHCs. In DHs, no clear trend was observed between the structural capacity index and quality of care as well as staffing and structural capacity index. However, a positive relationship between the quality of care index and staffing index was evident (**figure 3**).

DISCUSSION

This study provides evidence from all DHs and a large representative sample of block PHCs in Bihar, describing the gaps that need to be addressed to improve the provision of facility-based maternal and newborn care. Gaps in the structural capacity of facilities to provide quality care in terms of basic infrastructure, availability of equipment and supplies, and appropriate staffing were identified. These are areas that will require coordinated and dedicated efforts if much needed gains are to be made towards improved quality of facility-based maternal and neonatal care.

The results revealed that DHs, on average, were better in terms of staffing and structural capacity in comparison with PHCs. However, the reported quality of care was better in PHCs than the DHs. DHs, being the referral points for PHCs, often need to address complicated cases and are therefore recommended to have higher staffing and structural capacity in comparison to PHCs. However, the quality of care provided at DHs and PHCs would also depend on other factors including the case load and type of cases.

The trends within the structural capacity were very similar in both the district and block facilities with availability of supplies being the lowest amongst the components of structural capacity. It is particularly concerning that DHs are missing drugs to control blood pressure and treat haemorrhage since they are supposed to deal with women who are at risk of complications.

Maintenance of hygiene is extremely important in clinical areas such as labour rooms with patients at high risk of acquiring infections. However, assessment of infrastructure readiness revealed a low level of hygiene and sanitation practices in the facilities. The study identified lack of recommended handwashing stations in different rooms and colour coded bins in the labour room. The establishment of a system of accreditation and regular monitoring of quality of hygienic care, among other interventions, may help to ensure that the facilities have the essential equipment and infrastructure in place.

The most commonly missing equipment in the DHs and PHCs were mostly pertaining to neonatal care. Access to basic neonatal care is essential to reduce neonatal mortality because between a quarter and half of all neonatal deaths happen within 24hrs of life and 75% of neonatal deaths arise in the first week of life.[27] Pre-term birth, severe infections and asphyxia have been globally identified as the main direct causes of neonatal death. Low birth weight has also been recognized as an important cause of death.[27] Low-cost interventions including tetanus toxoid vaccination, exclusive breastfeeding, kangaroo mother care for low birthweight infants, and antibiotics for neonatal infections can reduce mortality.[28] However, our study revealed that skin to skin care was only being practiced by 36% of the mothers in PHCs and 24% of the mothers in DHs across Bihar. Immediate breastfeeding practice was also reported by only 24% of the mothers in PHCs

For peer review only - http://bmjopen.bmj.com/site/about/guidelines.xhtml

and 21% mothers DHs. These findings suggest that these facilities are not ready to provide quality neonatal care and are missing simple but vital life-saving interventions.

Availability of skilled human resources is another important aspect to provide quality maternal and newborn care. The data on sanctioned posts, as reported by the facility in charge, were found to be different from those recommended by IPHS guidelines. This gap between the guidelines and actual sanctioned posts reflects the lack of translation of policies into practice. In PHCs, while the essential requirement for MOs was fulfilled in all facilities, the filled to sanctioned ratio was only 68%, indicating the need for more MOs in these facilities. In DHs, the IPHS requirement of staff nurse/ANM was fulfilled in only 15% of the facilities. In addition, lack of staff was reported as the main reason for the unavailability of services. Our results specifically indicated a lack of counsellors at both the block and district facilities. This may have contributed to less than 30% mothers reporting having received advice about their and their baby's health before discharge. The positive relationship found between the staffing and quality of care (as reported by mothers) at the DHs affirms the need to address the gaps in staffing to provide better quality of care.

Both DHs and PHCs are particularly important platforms under the health system, with DHs being the secondary referral level responsible for providing basic specialty services and PHCs being the first point of contact to a qualified doctor in the public health sector in rural areas. Given that the PHCs are not equipped to manage complicated cases, including caesarean sections or provide facilities of SNCU, it is important to have a well-functioning transport system for referrals. Our findings revealed that 34% of the PHCs did not have an emergency transport for referrals. While the Government of India recommends the provision of referral system at the facilities, no systematic step has been taken in this direction so far.[26,29] Lack of skilled staff, inadequate infrastructure and lack of accountability have been recognized as some of the key reasons for the failure of referral systems in India.[30]

This study has both strengths and limitations. The study draws on data from a large number of facilities, covering all DHs and a large representative sample of PHCs in Bihar. To our knowledge, no study of this scale has previously been conducted on facility readiness for maternal and newborn care in Bihar. The findings of this study are, however, based on data collected from a single visit to these facilities; the availability of different equipment and supplies might vary over time. The number of responses varied across and within the components of infrastructure, supplies, equipment and staffing, leading to incomplete data in some facilities. With respect to household data, women providing information on quality of care were not representative of those delivering at facilities and the sampling was not proportional to the number of deliveries at each facility. Hence, findings on quality of care at facilities as reported in the household survey should be treated as exploratory findings only. The scope of this study is limited to assessing the structural capacity of the facilities to deliver quality care and the care as reported by the mothers. However, there could be multiple other components that influence quality of care (e.g. skills and competencies of health personnel delivering care) that were not explored in this study.

CONCLUSION

Presence of well-functioning facilities, with required structural and staffing capacity, is crucial for providing maternal and newborn care that translates to better maternal and child outcome. Being a highly populated state with poor health outcomes, the state of Bihar requires particular attention if India is to achieve the sustainable development goals targets for maternal and newborn health.

For peer review only - http://bmjopen.bmj.com/site/about/guidelines.xhtml

This study provides a description of the current capacity of public facilities in Bihar to provide quality maternal and neonatal care, unearthing particular gaps in neonatal equipment, infrastructure required to maintain hygiene, and staffing capacity at the facilities. Lack of correlation between structural capacity and staffing, and structural capacity and quality of care suggests presence of heterogeneity in the strengths and weaknesses across the facilities. A better understanding is needed to assess the cause of this variation which could help design tailored and appropriate interventions at these facilities to improve quality of care. This study lays the foundation for ongoing studies in Bihar to explore the relationship between quality of care and health outcomes. Increased focus on effective coverage and quality of facility-based care for mothers and newborns is needed if necessary gains are going to be made in saving lives in this high-burden setting.

Acknowledgements

The authors thank Sneha Lamba, Rakesh Chandra, Madhavi Misra, Tanmay Mahapatra and the entire field Team of OPM for their contribution to this manuscript.

Contributor ship Statement

T.NL. and S.F. conceived the idea for the manuscript. J.K. managed the data collection and supervised the data cleaning. J.K. led the data analysis and wrote the manuscript with the support of G.M. J.K. and G.M. jointly interpreted the data. G.M. supervised and edited the manuscript. Both T.NL. and S.F. reviewed the paper. All authors were involved in the interpretation of data, critically reviewed the manuscript and approved the final version.

Competing Interests

None declared.

Funding

This study was supported by Bill and Melinda Gates Foundation.

Data sharing Statement

Data used for this manuscript will be made available on request.

For peer review only - http://bmjopen.bmj.com/site/about/guidelines.xhtml

References

1. WHO, UNICEF, UNFPA, World Bank Group and UNPD. Maternal mortality in 1990-2015. 2015.

2. Lim SS, Dandona L, Hoisington JA, James SL, Hogan MC, Gakidou E. India's Janani Suraksha Yojana, a conditional cash transfer programme to increase births in health facilities: an impact evaluation. Lancet [Internet]. 2010;375(9730):2009–23. Available from: http://dx.doi.org/10.1016/S0140-6736(10)60744-1

3. Wang H, Bhutta ZA, Coates MM, Coggeshall M, Dandona L, Diallo K, et al. Global, regional, national, and selected subnational levels of stillbirths, neonatal, infant, and under-5 mortality, 1980–2015: a systematic analysis for the Global Burden of Disease Study 2015. Lancet. 2016;388(10053):1725–74.

4. Sustainable Development Knowledge Platform.

5. Government of India Planning Commission. Press note on poverty estimates, 2011-12. Press Information Bureau. 2013.

6. GoI. Sample Registration System Statistical Report 2016. 2017.

7. GoI. Sample Registration System Statistical Report 2012 [Internet]. New Delhi; 2013. Available from: http://www.censusindia.gov.in/vital_statistics/SRS_Reports_2012.html

8. National Family Health Survey - 4. 2015;

9. International Institute for Population Sciences. State Fact Sheet Bihar [Internet]. National Family Health Survey-4. 2015. Available from: http://rchiips.org/NFHS/pdf/NFHS4/BR_FactSheet.pdf

10. Bhutta ZA, Das JK, Bahl R, Lawn JE, Salam RA, Paul VK, et al. Can available interventions end preventable deaths in mothers, newborn babies, and stillbirths, and at what cost? Lancet. 2014;384(9940):347–70.

11. Das A, Rao D, Hagopian A. India's Janani Suraksha Yojana: Further review needed. Vol. 377, The Lancet. 2011. p. 295–6.

12. Gita G, Bharti S, Shashi K, Arvind K. Trends in maternal mortality and impact of janani suraksha yojana (JSY) on maternal mortality ratio in a tertiary referral hospital. J Obstet Gynecol India. 2012;62(3):307–11.

13. Jayanna K, Mony P, Ramesh BM, Thomas A, Gaikwad A, Mohan HL, et al. Assessment of facility readiness and provider preparedness for dealing with postpartum haemorrhage and pre-eclampsia/eclampsia in public and private health facilities of northern Karnataka, India: A cross-sectional study. BMC Pregnancy Childbirth. 2014;14(1).

14. Dickson KE, Simen-Kapeu A, Kinney M V., Huicho L, Vesel L, Lackritz E, et al. Every Newborn: Health-systems bottlenecks and strategies to accelerate scale-up in countries. Vol. 384, The Lancet. 2014. p. 438–54.

15. Rammohan A, Iqbal K, Awofeso N. Reducing Neonatal Mortality in India: Critical Role of Access to Emergency Obstetric Care. PLoS One. 2013;8(3).

16. Ramarao S. Safer maternal health in rural Uttar Pradesh: do primary health services

For peer review only - http://bmjopen.bmj.com/site/about/guidelines.xhtml

contribute? Health Policy Plan. 2002;16(3):256–63.

17. Moxon SG, Lawn JE, Dickson KE, Simen-Kapeu A, Gupta G, Deorari A, et al. Inpatient care of small and sick newborns: A multi-country analysis of health system bottlenecks and potential solutions. BMC Pregnancy Childbirth. 2015;15.

18. Sharma G, Mathai M, Dickson KE, Weeks A, Hofmeyr GJ, Lavender T, et al. Quality care during labour and birth : a multi- country analysis of health system bottlenecks and potential solutions. BMC Pregnancy Childbirth [Internet]. 2015;15(Suppl 2):S2. Available from: http://www.biomedcentral.com/1471-2393/15/S2/S2

19. WHO. Standards for Improving Quality of Maternal and Newborn Care in Health Facilities [Internet]. Who. 2016. Available from: http://apps.who.int/iris/bitstream/10665/249155/1/9789241511216-eng.pdf?ua=1

20. Chaturvedi S, De Costa A, Raven J. Does the Janani Suraksha Yojana cash transfer programme to promote facility births in India ensure skilled birth attendance? A qualitative study of intrapartum care in Madhya Pradesh. Glob Health Action. 2015;8(1).

21. Ministry of Health and Family Welfare, GoI. Rural Health Statistics, 2014-15. 2015.

22. CARE. Bihar Technical Support Programme - CARE India [Internet]. 2017. Available from: https://www.careindia.org/project/technical-assistance-to-the-government-of-bihar-improving-health-nutrition-coverage-and-outcomes/

23. Das A, Chatterjee R, Karthick M, Mahapatra T, Chaudhuri I. The influence of seasonality and community-based health worker provided counselling on exclusive breastfeeding - Findings from a cross-sectional survey in India. Vol. 11, PLoS ONE. 2016.

24. WHO. Service Availability and Readiness Assessment (SARA) [Internet]. World Health Organisation (WHO). 2016. Available from: http://www.who.int/healthinfo/systems/sara_indicators_questionnaire/en/

25. Services DG of H, India M of H& FWG of. Indian Public Health Standards (Iphs) Guidelines for Primary Health Centres Revised 2012 [Internet]. Directorate General of Health Services Ministry of Health & Family Welfare Government of India. 2012. Available from: http://health.bih.nic.in/Docs/Guidelines/Guidelines-PHC-2012.pdf

26. Ministry of Health & Family Welfare. Indian Public Health Standards (IPHS) Guidelines for District Hospitals (101 to 500 Bedded [Internet]. 2012. Available from: http://nrhm.gov.in/images/pdf/guidelines/iphs/iphs-revised-guidlines-2012/district-hospital.pdf

27. Lawn JE, Cousens S, Zupan J. 4 Million neonatal deaths: When? Where? Why? Vol. 365, Lancet. 2005. p. 891–900.

28. Martines J, Paul VK, Bhutta ZA, Koblinsky M, Soucat A, Walker N, et al. Neonatal survival: A call for action. Vol. 365, Lancet. 2005. p. 1189–97.

29. Vora KS, Mavalankar D V., Ramani K V., Upadhyaya M, Sharma B, Iyengar S, et al. Maternal health situation in India: A case study. J Heal Popul Nutr. 2009;27(2):184–201.

30. Anil Kumar Gupta, Shweta Talati, Sudip Bhattacharya AS. Health System Strengthening-Focussing on Referrals: An Analysis from India. JOJ Nurs Heal care. 2017;2(4).

For peer review only

For peer review only - http://bmjopen.bmj.com/site/about/guidelines.xhtml

Figure Legends

Figure 1: Reasons for unavailability of services in (A) primary health centres (PHCs) and (B) district hospitals (DHs). Multiple answers were allowed. Figures in parentheses of x axis report the total unavailability Abbreviations: MTP: Medical termination of pregnancy; ECP: Emergency contraceptive pill; VDRL: Venereal Disease Research Laboratory; PPIUCD: Postpartum Intrauterine Contraceptive Device; ANC: Ante natal care

Figure 2: Structural readiness scores across district hospitals (DHs) and primary health centres (PHCs). Scores are presented as box plots representing the median and interquartile range (box and whiskers, respectively) and outliers (dots).

Figure 3: Structural capacity, staffing and quality of care relationship for primary health centres (blue) and district hospitals (orange). Each point represents the indexes for each facility (PHC or DH). The trend line shows the relationship between the staffing and quality of care across the PHCs (blue) and DHs (orange). Note that axis scales vary.

For peer review only

Figure 1: Reasons for unavailability of services in (A) primary health centres (PHCs) and (B) district hospitals (DHs)

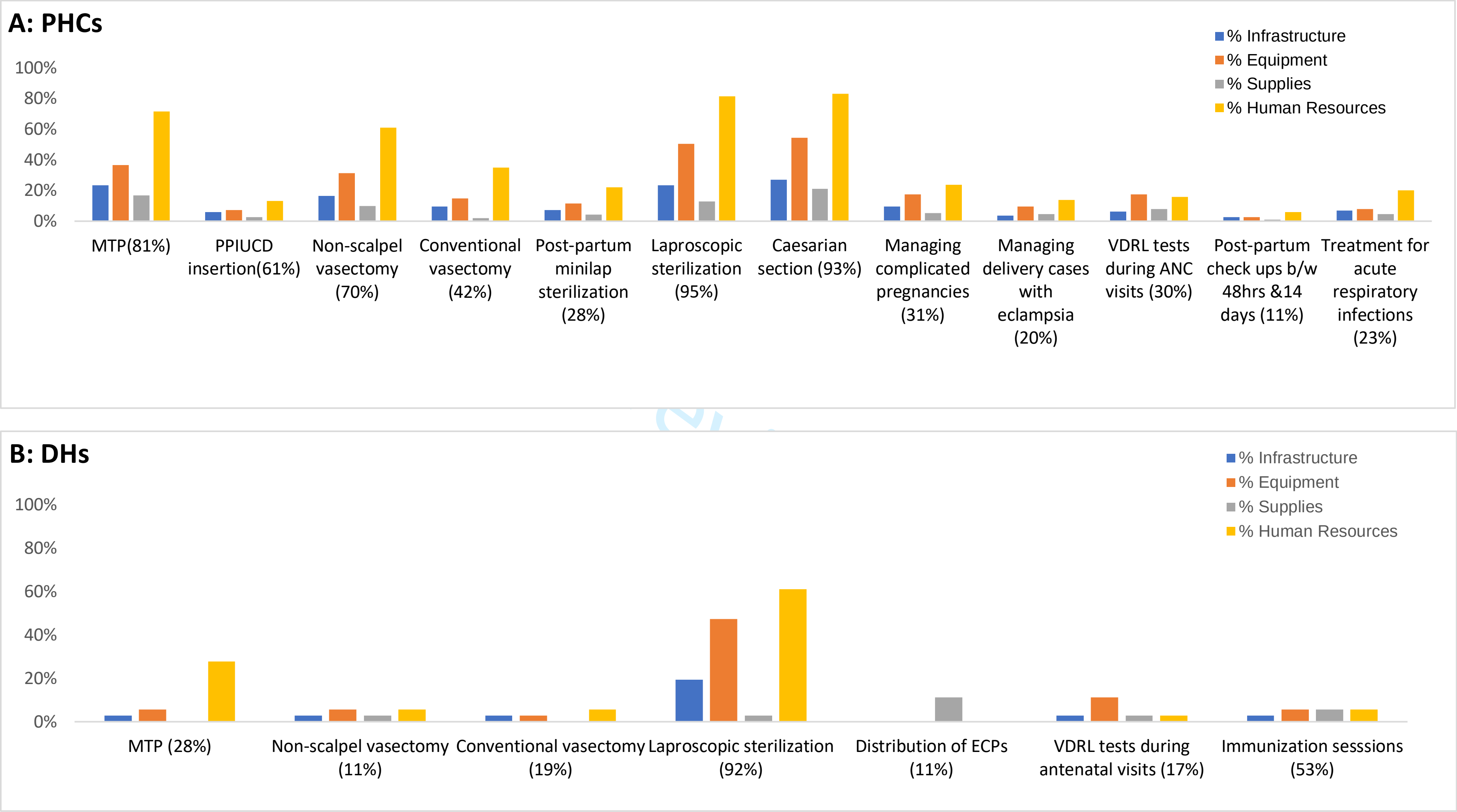

Multiple answers were allowed. Figures in parentheses of x axis report the total unavailability

Abbreviations: MTP: Medical termination of pregnancy; ECP: Emergency contraceptive pill; VDRL: Venereal Disease Research Laboratory; PPIUCD: Postpartum Intrauterine Contraceptive Device; ANC: Ante natal care

Figure 2: Structural readiness scores across district hospitals (DHs) and primary health centres (PHCs)

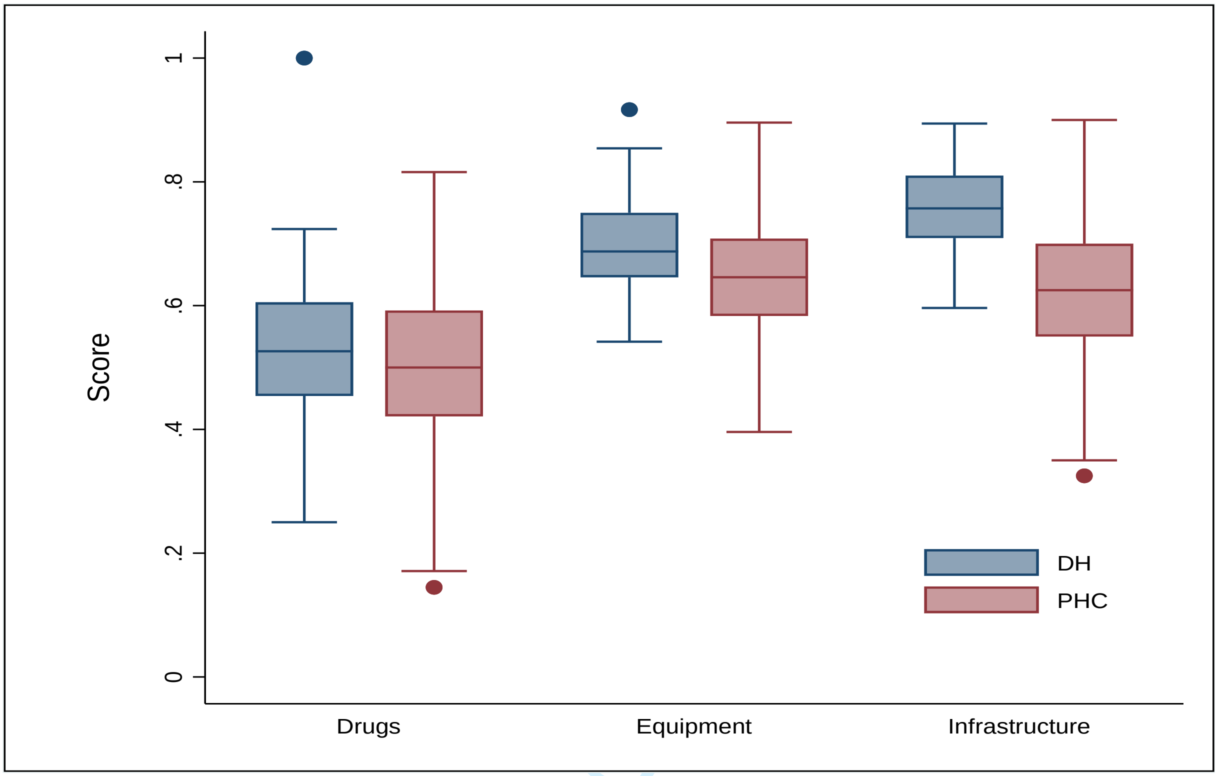

Scores are presented as box plots representing the median and interquartile range (box and whiskers, respectively) and outliers (dots).

Figure 3: Structural capacity, staffing and quality of care relationship for primary health centres (blue) and district hospitals (orange)

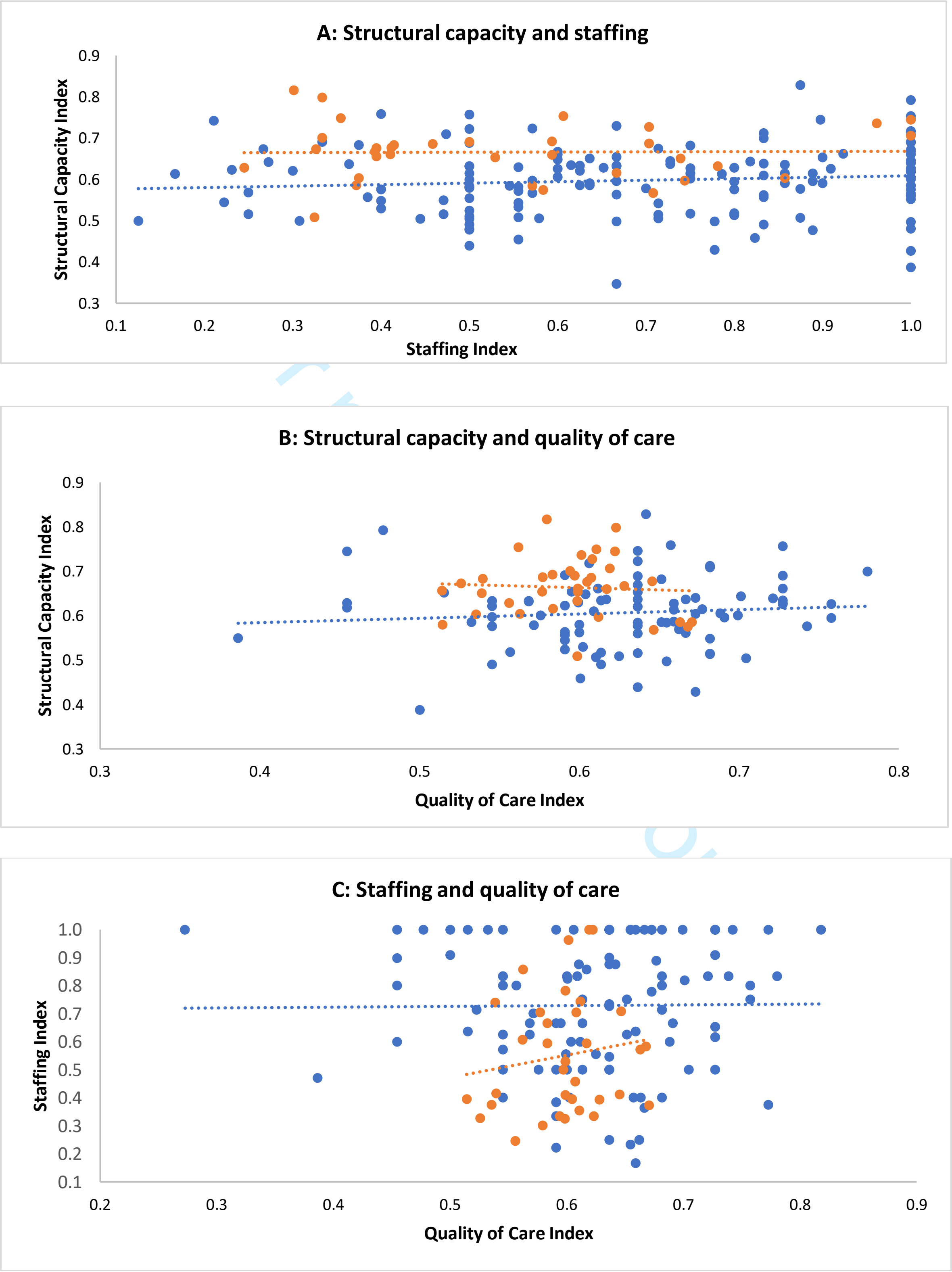

Each point represents the indexes for each facility (PHC or DH). The trend line shows the relationship between the staffing and quality of care across the PHCs (blue) and DHs (orange). Note that axis scales vary.

APPENDIX 1

Additional methods

Infrastructure scoring

A score out of 1 was generated for each of these components. These scores were added, giving equal weight to each component and divided by the total components to generate an overall score ranging from 0 to 1, which was then converted to a percentage by multiplying by 100.

To compute a score for the component 'power/electricity' in the facilities, a value 1 was assigned to the facilities with continuous power, 0.75 to the facilities which reported occasional disruption to power in summer only, 0.5 to the facilities that were facing occasional disruption to power throughout the year, 0.25 to facilities with regular interruption to power and 0 to the facilities that reported no power supply. In cases where back-up was available that met the entire needs of the facility the above score was replaced by 1. For the 'water' score, 0.5 was assigned for availability of 24*7 running water and 0.5 if filtered water was available to patients. For telephone connection, a value 1 was assigned if it was available, else 0.

The transport score was computed by giving a score 0.5 to availability of at least one functional ambulance and an additional 0.5 to 24*7 availability. For toilets, 0.5 was assigned in case a functional toilet was available and 0.25 in case a toilet was available but not functional. Another 0.5 was assigned if the condition of the toilet was good and 0.25 if it needed repair/maintenance. For handwashing stations, 1 was assigned for the availability for elbow tap with running water and 0.5 was assigned for tap (without elbow) with running water. For computer and internet, 0.5 was assigned if the hospital had provided a computer and an additional 0.5 if there was internet connection available at the facility. For blood bank, 1 was assigned if there was a blood bank provision at the hospital, else 0.

For room availability, a score of 1 was assigned for the availability of each of the 16 rooms and then, it was added across rooms and divided by 16 to get a score out of 1. The 16 rooms were waiting room, labour room, maternity ward, immunization room, counselling room, operation theatre, store room, paediatrics, labour room eclampsia, SNCU (sick new born care unit), NBSU (new born stabilization units), septic labour room, antenatal ward, postnatal ward, postpartum ward and post-operative ward.

Equipment scoring

To compute the equipment score, a value 1 was assigned to each equipment if it was available and functional, else it was assigned 0. After computing the score for each equipment, we divided it by 48 (total equipment) to get a score out of 1 and multiplied by 100 to get the percentage distribution.

Supplies/drugs scoring

The availability of a drug was assigned a value 1, else it was assigned 0. The scores for each of the drugs were added, divided by the total (76) and then multiplied by 100 to get the percentage distribution.

For peer review only

Table S1: Infrastructure assessed

Infrastructure Components	Facility Level
Handwashing in immunization Room	PHCs, DHs
Handwashing in laboratory	PHCs, DHs
Handwashing in operation theatre	PHCs, DHs
Toilet in labour room	PHCs, DHs
Handwashing in labour room	PHCs, DHs
Transport	PHCs, DHs
Water	PHCs, DHs
Power	PHCs, DHs
Phone	PHCs, DHs
Room availability	DHs
Blood bank	DHs
Computer and internet	DHs

Abbreviations: PHC: Primary health centre, DH: District hospital

Table S2: Equipment assessed in district hospitals and primary health centres

Equipment
Caserean kit
Neonatal stethoscope
MTP suction
Light examination
NSV kit
Infantometer
Baby incubator
MTP kit
Nebulizer
Feeding tube
Shadowless lamp (OT)
Conventional vasectomy kit
Oxygen cylinder (LR)
Minilap kit
Resuscitation kit
Spotlight (OT)
Autoclave (NBCC)
Stethoscope (OT)
IV stand (OT)
Step up stool
Immunization table
Oxygen cylinder (OT)
Hub cutter (immunization room)
Partograph
Phototherapy unit

Cord clamp
Thermometer (LR)
IV cannula
Hub cutter (NBCC)
PPIUCD kit
Pump suction
Autoclave (OT)
Freeze tags
Stethoscope (LR)
Sterile gloves
Radiant warmer
Thermometer (immunization room)
Delivery kit
IUCD kit
Refrigerator
Ambu bag (NBCC)
Deep freezer
Operating table
Vaccine Carrier
BP Instrument
Labour table
Baby weighing machine
Ice packs

Information in parantheses represent the room in which the equipment was checked.

Abbreviations: MTP: Medical termination of pregnancy; NSV: Non-scalpel vasectomy; OT: Operation theatre; LR: labour room; NBCC: Newborn care corner; PPIUCD: Postpartum Intrauterine Contraceptive Device; IUCD: Intra-Uterine Contraceptive Devices; BP: Blood pressure

Table S3: Drugs assessed in district hospitals and primary health centres

Drugs
Amitriptyline
Captopril
Suxamethonium bromide
Mephenteramine
Carboprost
Miltefosine
Syrup salbutamol
Hydralazine
Omeprazole
Chlorine solution
Halothane (inhalation)
Menadione (Vit K3)
IUCD 375
Methyldopa
Simvastatin
Amphotericin B
Liposomal amphotericin B
Phytonadione
Glibenclamide
Skin disinfectant
Vitamin K
Thiopental (powder)
Dopamine
OPV
Deriphylline
BCG
Hepatitis B
DPT
Pentavalent
Frusemide
Measles
Epinephrine
Syrup vitamin A
Iron folic acid – small
Atenolol
Pentazocin chloride
Ceftriaxone
Methylegometrine (Methargine)
Nifedepine
Xylocard
Promethazine
Betamethasone
Hydrocortisone succinate

Antiseptic solution
Tetanus Toxoid
Pheneramine maleate
Adrenaline
Co-trimoxazole
Injection ranitidine
Zinc tablets
Syrup paracetamol
IUCD 380A
DEC (Diethyl Carbamazine)
Iron folic acid – Large
Amoxicillin
Metoclopramide
OCPs (Mala-N)
Ampicillin
Calcium gluconate
ECPs
Lignocaine hydrochloride 2%
Misoprostol
Condoms (Nirodh)
Ciprofloxacin
Diclofenac
Magnesium sulphate
Gentamycin
Atropine
Oxytocin
Albendazole
Ketamine
Diazepam
ORS
Dexamethasone
Metronidazole
Paracetamol

Abbreviation: IUCD: Intra-Uterine Contraceptive Devices; BCG: Bacille Calmette Guerin; DPT: Diphtheria, Pertussis and tetanus; OPV: Oral Polio Vaccine; ORS: Oral Rehydration Solution; OCP: Oral contraceptive pill; ECP: Emergency contraceptive pill

Table S4: Essential and Desirable Staff Guidelines at district hospitals (DHs) and primary health centres (PHCs) as per IPHS Guidelines

A: PHCs

IPHS Guidelines	Type A		Type B	
Staff	Essential	Desirable	Essential	Desirable
Medical Officer-MBBS	1		1	1
Medical Officer-AYUSH		1		1
Pharmacist	1		1	
Nurse-midwife (ANMs)	3	1	4	1
Laboratory Technician	1		1	

Type A PHC: PHC with delivery load of less than 20 deliveries in a month and Type B PHC: PHC with delivery load of 20 or more deliveries in a month

B: DHs

IPHS Requirements	100 Beds	200 Beds	300 Beds	400 Beds	500 Beds
Medical officer	11	13	15	19	23
Staff Nurse/ANMs	45	90	135	180	225
Lab Technician	6	9	12	15	18
Compounder/Pharmacist	5	7	9	11	13
Store Keeper	1	1	2	2	2

Table S5: Number of respondents at district hospitals (DHs) and primary health centres (PHCs).

No. of respondents at DHs	No. of respondents at PHCs	Information covered
36	190	Overall Infrastructure, Staffing
35	171	Laboratory: Infrastructure
36	179	Labour room: Infrastructure, Equipment, Supplies
35	181	Immunization room: Infrastructure, Equipment
36	166	Operation Theatre: Infrastructure, Equipment
35	177	Drug Store room: Supplies

Note: This table shows the number of respondents that were available for each of these components. The information on sub-components could vary based on the knowledge of the respondent.

Table S6: Reasons for unavailability of services at district hospitals (DHs) and primary health centres (PHCs)

	% Unavailable		*Reasons for unavailability*							
			Infrastructure		Equipment		Drugs		Supplies	
Services	DHs	PHCs	DHs	PHCs	DHs	PHCs	DHs	PHCs	DHs	PHCs
MTP	28%	81%	3%	23%	6%	37%	0%	17%	28%	71%
IUCD insertion	0%	3%	0%	1%	0%	1%	0%	0%	0%	2%
IUCD removal	0%	3%	0%	1%	0%	1%	0%	0%	0%	2%
Post-partum IUCD insertion	0%	16%	0%	6%	0%	7%	0%	3%	0%	13%
Non-scalpel vasectomy	11%	70%	3%	16%	6%	31%	3%	10%	6%	61%

For peer review only

Conventional vasectomy	19%	42%	3%	10%	3%	15%	0%	2%	6%	35%
Minilap tubectomy	3%	8%	3%	2%	0%	2%	0%	0%	3%	7%
Post-partum minilap sterilization	3%	28%	0%	7%	3%	12%	0%	4%	3%	22%
Laparoscopic sterilization	92%	95%	19%	23%	47%	50%	3%	13%	61%	81%
Distribution of condoms	6%	3%	0%	0%	0%	0%	3%	1%	0%	2%
Distribution of OCPs	3%	3%	0%	0%	0%	0%	3%	1%	0%	2%
Distribution of ECPs	11%	6%	0%	1%	0%	0%	11%	2%	0%	3%
Normal delivery	0%	5%	0%	2%	0%	2%	0%	1%	0%	3%
Caesarian section	3%	-	3%	-	3%	-	0%	-	0%	-
Managing complicated pregnancies	0%	31%	0%	10%	0%	17%	0%	5%	0%	24%
Managing delivery cases with eclampsia	8%	20%	0%	4%	0%	10%	0%	5%	8%	14%
Registration of pregnancies	0%	2%	0%	0%	0%	1%	0%	0%	0%	1%
Antenatal care visits	0%	2%	0%	0%	0%	1%	0%	0%	0%	1%
Distribution of IFA tablets	0%	5%	0%	0%	0%	1%	0%	4%	0%	1%
Tetanus toxoid vaccinations	3%	1%	3%	0%	0%	1%	0%	0%	0%	1%
VDRL tests during antenatal visits	17%	30%	3%	6%	11%	17%	3%	8%	3%	16%
BP tests during antenatal visits	0%	1%	0%	0%	0%	1%	0%	0%	0%	1%
HB tests during antenatal visits	0%	8%	0%	1%	0%	5%	0%	4%	0%	5%
Weighing of newborns at birth	0%	4%	0%	2%	0%	2%	0%	1%	0%	3%
Neonatal resuscitation in cases with asphyxia	3%	7%	3%	3%	0%	5%	0%	2%	3%	6%
Post-partum check ups within 48hrs of delivery	0%	5%	0%	2%	0%	2%	0%	1%	0%	4%
Post-partum check ups b/w 48hrs &14 days of delivery	0%	11%	0%	3%	0%	3%	0%	1%	0%	6%
Immunization sessions	53%	2%	3%	0%	6%	1%	6%	0%	6%	2%
Treatment for diarrhea	0%	3%	0%	1%	0%	2%	0%	1%	0%	2%
Treatment for acute respiratory infections	8%	23%	3%	7%	3%	8%	0%	5%	3%	20%

Abbreviations: MTP: Medical termination of pregnancy; IUCD: Intra-Uterine Contraceptive Devices; OCP: Oral contraceptive pill; ECP: Emergency contraceptive pill; IFA: Iron folic acid

For peer review only

Table S7: Most commonly missing and available structural capacity items across primary health centres and district hospitals

	Infrastructure		Equipment		Drugs	
	Most commonly missing	Most commonly available	Most commonly missing	Most commonly available	Most commonly missing	Most commonly available
Primary health centres	Handwashing in immunization room (13%)	Water (81%)	Neonatal stethoscope (16%)	Deep freezer, Ambu bag (97%)	Amitriptyline (12%)	ORS (95%)
	Handwashing in laboratory (37%)	Power (87%)	MTP suction (17%)	Operating table, labour table, BP Instrument, vaccine carrier (98%)	Captopril (14%)	Dexamethasone (99%)
	Handwashing in OT (60%)	Phone (93%)	Light examination (20%)	Baby weighing machine, ice packs (99%)	Suxamethonium bromide, mephenteramine (16%)	Paracetamol, metronidazole (100%)
District hospitals	Handwashing in immunization room (19%)	Water (97%)	Infantometer, nebulizer, baby incubator (14%)	IV cannula, PPIUCD kit, ice packs (94%)	Captopril, phytanodiane, simvastin, IUCD 35 (3%)	Atropine, DPT (94%)
	Handwashing in laboratory (47%)	Power (99%)	Neonatal stethoscope, MTP suction (39%)	Vaccine carrier, operating table, delivery kit, oxygen cylinder (97%)	Amritriptyline (6%)	Dexamethasone, metronidazole, paracetamol, BCG, OPV, measles, pentavalent (97%)
	Room Availability (64%)	Transport (100%)	Light examination, resuscitation kit, phototherapy unit (42%)	Labour table, MTP kit, IUCD kit, Ambu bag, IV stand (100%)	Glibenclamide, suxamethonium bromide, syrup vit A (8%)	Diazepam, tetanus toxoid (100%)

For peer review only

STROBE Statement—Checklist of items that should be included in reports of ***cross-sectional studies***

	Item No	Recommendation	Page No
**Title and abstract**	1	(*a*) Indicate the study's design with a commonly used term in the title or the abstract	1
		(*b*) Provide in the abstract an informative and balanced summary of what was done and what was found	1
**Introduction**			
Background/rationale	2	Explain the scientific background and rationale for the investigation being reported	2,3
Objectives	3	State specific objectives, including any prespecified hypotheses	3
**Methods**			
Study design	4	Present key elements of study design early in the paper	3
Setting	5	Describe the setting, locations, and relevant dates, including periods of recruitment, exposure, follow-up, and data collection	3
Participants	6	(*a*) Give the eligibility criteria, and the sources and methods of selection of participants	4
Variables	7	Clearly define all outcomes, exposures, predictors, potential confounders, and effect modifiers. Give diagnostic criteria, if applicable	5,6
Data sources/ measurement	8*	For each variable of interest, give sources of data and details of methods of assessment (measurement). Describe comparability of assessment methods if there is more than one group	*4,5*
Bias	9	Describe any efforts to address potential sources of bias	2
Study size	10	Explain how the study size was arrived at	3,4
Quantitative variables	11	Explain how quantitative variables were handled in the analyses. If applicable, describe which groupings were chosen and why	6
Statistical methods	12	(*a*) Describe all statistical methods, including those used to control for confounding	5,6
		(*b*) Describe any methods used to examine subgroups and interactions	6
		(*c*) Explain how missing data were addressed	6
		(*d*) If applicable, describe analytical methods taking account of sampling strategy	
		(*e*) Describe any sensitivity analyses	
**Results**			
Participants	13*	(a) Report numbers of individuals at each stage of study—eg numbers potentially eligible, examined for eligibility, confirmed eligible, included in the study, completing follow-up, and analysed	6
		(b) Give reasons for non-participation at each stage	6
		(c) Consider use of a flow diagram	
Descriptive data	14*	(a) Give characteristics of study participants (eg demographic, clinical, social) and information on exposures and potential confounders	6,7
		(b) Indicate number of participants with missing data for each variable of interest	6
Outcome data	15*	Report numbers of outcome events or summary measures	6
Main results	16	(*a*) Give unadjusted estimates and, if applicable, confounder-adjusted estimates and their precision (eg, 95% confidence interval). Make clear which confounders were adjusted for and why they were included	7-10

For peer review only - http://bmjopen.bmj.com/site/about/guidelines.xhtml

		(*b*) Report category boundaries when continuous variables were categorized	
		(*c*) If relevant, consider translating estimates of relative risk into absolute risk for a meaningful time period	
Other analyses	17	Report other analyses done—eg analyses of subgroups and interactions, and sensitivity analyses	7-10
**Discussion**			
Key results	18	Summarise key results with reference to study objectives	11,12
Limitations	19	Discuss limitations of the study, taking into account sources of potential bias or imprecision. Discuss both direction and magnitude of any potential bias	12
Interpretation	20	Give a cautious overall interpretation of results considering objectives, limitations, multiplicity of analyses, results from similar studies, and other relevant evidence	11,12
Generalisability	21	Discuss the generalisability (external validity) of the study results	12
**Other information**			
Funding	22	Give the source of funding and the role of the funders for the present study and, if applicable, for the original study on which the present article is based	13

*Give information separately for exposed and unexposed groups.

Note: An Explanation and Elaboration article discusses each checklist item and gives methodological background and published examples of transparent reporting. The STROBE checklist is best used in conjunction with this article (freely available on the Web sites of PLoS Medicine at http://www.plosmedicine.org/, Annals of Internal Medicine at http://www.annals.org/, and Epidemiology at http://www.epidem.com/). Information on the STROBE Initiative is available at www.strobe-statement.org.

For peer review only
